# Synthesis and Biological Properties of EGFR-Targeted Photosensitizer Based on Cationic Porphyrin

**DOI:** 10.3390/pharmaceutics15041284

**Published:** 2023-04-19

**Authors:** Yulia S. Bortnevskaya, Nikita A. Shiryaev, Nikita S. Zakharov, Oleg O. Kitoroage, Margarita A. Gradova, Natalia Yu. Karpechenko, Alexander S. Novikov, Elena D. Nikolskaya, Mariia R. Mollaeva, Nikita G. Yabbarov, Natal’ya A. Bragina, Kseniya A. Zhdanova

**Affiliations:** 1 Institute of Fine Chemical Technology, MIREA—Russian Technological University, Vernadsky pr., 86, 119571 Moscow, Russia; bortnevskaja2000@yandex.ru (Y.S.B.); lost.slippers@yandex.ru (N.A.S.); nikita_zakharov_0_0@mail.ru (N.S.Z.); kitoroage95@mail.ru (O.O.K.); n.bragina@mail.ru (N.A.B.); zhdanova_k@mirea.ru (K.A.Z.); 2 N. N. Semenov Federal Research Center for Chemical Physics, Russian Academy of Sciences, Kosygin St., 4, 119991 Moscow, Russia; m.a.gradova@gmail.com; 3 N. N. Blokhin National Medical Research Center of Oncology, Ministry of Health of Russia, Kashirskoe Highway, 24, 115522 Moscow, Russia; nojadg@mail.ru; 4 Department of Medical Chemistry and Toxicology, Pirogov National Research Medical University, Ministry of Health of Russia, Ostrovityanova St., 1, 117997 Moscow, Russia; 5 Institute of Chemistry, Saint Petersburg State University, Universitetskaya nab. 7–9, 199034 Saint Petersburg, Russia; 6 Research Institute of Chemistry, Peoples’ Friendship University of Russia (RUDN University), Miklukho-Maklaya St., 6, 117198 Moscow, Russia; 7 Emanuel Institute of Biochemical Physics, Russian Academy of Sciences, Kosygina St., 4, 119334 Moscow, Russia; elenanikolskaja@gmail.com (E.D.N.); mollaevamariia@gmail.com (M.R.M.); nikita_yabbarov@yahoo.com (N.G.Y.)

**Keywords:** PDT, antitumor efficacy, meso-arylporphyrins, erlotinib, conjugates, porphyrin metal complexes, Pluronic F127, polymer nanomicelles, nano-formulation

## Abstract

Photodynamic therapy (PDT) in oncology is characterized by low invasiveness, minimal side effects, and little tissue scarring. Increasing the selectivity of PDT agents toward a cellular target is a new approach intended to improve this method. This study is devoted to the design and synthesis of a new conjugate based on meso-arylporphyrin with a low-molecular-weight tyrosine kinase inhibitor, Erlotinib. A nano-formulation based on Pluronic F127 micelles was obtained and characterized. The photophysical and photochemical properties and biological activity of the studied compounds and their nano-formulation were studied. A significant, 20–40-fold difference between the dark and photoinduced activity was achieved for the conjugate nanomicelles. After irradiation, the studied conjugate nanomicelles were 1.8 times more toxic toward the EGFR-overexpressing cell line MDA-MB-231 compared to the conditionally normal NKE cells. The IC50 was 0.073 ± 0.014 μM for the MDA-MB-231 cell line and 0.13 ± 0.018 μM for NKE cells after irradiation for the target conjugate nanomicelles.

## 1. Introduction

The search for new strategies for the diagnosis and therapy of oncological diseases is currently an urgent task in the field of medicine. In this respect, the photodynamic therapy (PDT) of oncological diseases is a promising new method [1,2,3]. PDT is a non-invasive treatment method based on binary therapy wherein a photosensitizer (PS) accumulates in tumor tissue and, upon light irradiation, generates reactive oxygen species (ROS), resulting in the destruction of vital cell structures and cell death [4]. However, unrestrained tumor growth leads to hypoxia of the cancer cell microenvironment, which significantly limits PDT’s efficiency [5,6]. During PDT, intracellular oxygen consumption can further aggravate tumor hypoxia and lead to decreased PDT efficiency.

It is also known that under ROS-mediated damage, one of the agents of cell survival, namely, hypoxia-inducible transcription factor (HIF-1), can be additionally activated [7,8]. HIF-1 induces the production of peptide hormones such as vascular endothelial growth factor (VEGF), which stimulates accelerated blood vessel growth (angiogenesis) in tumors. At the same time, the generation of ROS during PDT triggers the overexpression of anti-apoptotic proteins to cope with induced cell apoptosis [9]. If the effects that promote cell survival prevail, the tumor will grow again and become resistant to the drug. Thus, the disruption of tumor cell survival mechanisms unlocks another method with which to improve PDT efficacy. The use of VEGF receptor inhibitors overexpressed in cancer cells is one such method. To date, histological studies have indicated that the epidermal growth factor receptor (EGFR) family, including four transmembrane tyrosine kinases, is overexpressed in many tumor tissues [10]. Since high expression and functional activation of EGFR correlates with the pathogenesis of some cancers, EGFR can serve as an attractive target for both the diagnosis and therapy of neoplasia [11,12,13]. The use of drugs based on low-molecular-weight tyrosine kinase inhibitors (TKIs) that bind to the catalytic site of intracellular EGFR has been proposed [14]. Monoclonal antibodies (mAb) that act on the extracellular domains of a receptor, thereby blocking its dimerization, and peptides, antibodies, and vaccines that directly inhibit EGF have all proven effective [15,16,17,18]. However, low-molecular-weight TKI inhibitors are even more interesting. More than 40 clinically approved compounds are currently known [18]. Reversible first-generation EGFR tyrosine kinase inhibitors (gefitinib and erlotinib) have clinically improved prognosis in patients with non-small-cell lung cancer (NSCLC), medullary thyroid carcinoma, and breast cancer [19,20,21]. To date, the use of many of the studied photosensitizers is limited by their lack of selective targeting in tumor cells, which leads to the damage of healthy tissues and, therefore, introduces significant obstacles to the use of PDT. The creation of conjugates of low-molecular-weight tyrosine kinase inhibitors and photosensitizers can improve the PDT of cancer.

Porphyrins and their derivatives are among the most widely studied PSs for PDT applications (e.g., with respect to their intense absorption in the visible and near-infrared regions, fluorescent properties, efficient generation of singlet oxygen, and high quantum yield of ROS) [22,23,24]. The use of synthetic porphyrins offers several advantages, including low cost, high possibility for functionalization, a high quantum yield of ROS, and stability [25]. However, the hydrophobic character of porphyrins’ cores limits their use. The encapsulation of tetrapyrrole molecules into delivery systems has enhanced their therapeutic efficacy. Polymer micelles have received a great deal of attention with respect to many existing nanocarriers because of their simple preparation, remarkable solubilizing properties, and biocompatibility [26,27,28]. Pluronic F127 micelles consist of polyethylene oxide (PEO) and polypropylene oxide (PPO) blocks. Recently, these micelles were approved for clinical use [29,30]. In a recent study, we showed the efficiency of Pluronic F127 micelles with respect to the solubilization of cationic porphyrins and studied their antibacterial activity [31].

Earlier, new PSs based on chlorine e6 phthalocyanines and erlotinib were proposed [32,33]. Due to the introduction of erlotinib fragments into different structural positions of the PS macrocycle, the conjugates showed high specificity toward HepG2 cells and A431 tumor tissues and presented increased photodynamic activity against neoplasia compared to unsubstituted PS derivatives [34,35]. However, in these studies, nanocarriers were not used. 

In this work, we developed a synthetic strategy for the reception of new hybrid photosensitizers based on cationic porphyrins using erlotinib, which provides directional transport of PS to the target. Nanoscale delivery systems based on Pluronic F127 micelles were used. The photochemical and photophysical properties of the obtained PS were studied along with their photodynamic activity.

## 2. Materials and Methods

### 2.1. Chemistry

In this study, commercially available reagents were used. The solvents were purified according to known methods. Electronic absorption spectra of porphyrins were obtained using a HACH DR-4000V (Hach-Lange, Ames, IA, USA) spectrophotometer operating in 320–800 nm spectral range with 10 mm quartz cells at room temperature. Stationary fluorescence spectra were recorded using a Perkin Elmer LS-50 fluorescence spectrometer (Perkin Elmer, Waltham, MA, USA) under similar conditions at a monochromator slit width of 10 nm and an excitation wavelength corresponding to the Sore band maximum. NMR spectra were obtained on a BrukerMSL-300 pulsed Fourier spectrometer (FRG) with an operating frequency of 300 MHz; measurements were performed on the δ scale using TMS as an internal standard and CDCl_3_ or (CD_3_)_2_SO as a solvent. Tetramethylsilane was used as an external standard. Mass spectra were recorded using a matrix-activated laser desorption/ionization time-of-flight mass spectrometer (MALDI-TOF), for which the matrix used was 3,5 dihydroxybenzoic acid. HPLC MSWP analysis was performed on a Vanquish ultra-high-performance liquid chromatographic system coupled with a Q-Exactive HF-X high-resolution hybrid mass spectrometer under electrospray ionization. The individuality of the obtained compounds was determined by TLC on TLC Silicagel 60 F254 plates (Merck, Rahway, NJ, USA). Silica 60 silica gel 0.04–0.064 mm/230–400 mesh ASTM (Macherey-Nagel GmbH & Co. KG, Düren, Germany) was used for column chromatography.

#### 2.1.1. Synthesis of 4-(4-Bromo-n-butoxy)benzaldehyde **1**

A mixture of 4-hydroxybenzaldehyde (3 g, 24.6 mmol); 1,4-dibromobuthane (6.89 g, 31.9 mmol); and potassium carbonate (4.41 g, 3.9 mmol) was dissolved in 30 mL of acetone. The reaction occurred in a boiling liquid that was mechanically stirred for 12 h. Then, the reaction mixture was cooled and filtered. Extraction was performed in a CH_2_Cl_2_/H_2_O medium to which 1 M HCl was added until neutralization. The target product was purified using column chromatography on silica gel G60 (CH_2_Cl_2_:Hexane = 5:1 *v/v*). The yield was 4.52 g (76%). R_f_ = 0.4 (CH_2_Cl_2_:Hexane = 7:1 *v/v*). IR (ν, cm^−1^): 2928, 2849 (CH_2_)_4_, 1675 (C=O), 1467 (Ar), 1160, 1138 (C-O), 642, and 550 (C-Br). 1H-NMR (CDCl_3_, δ, ppm): 1.73 (2H, m, CH_2_CH_2_Br), 1.87 (2H, m, OCH_2_CH_2_), 3.39 (2H, t, *J* = 6.00 Hz, CH_2_CH_2_Br), 4.07 (2H, t, *J* = 6.00 Hz, OCH_2_), 7.51 (2H, d, *J* = 8.40 Hz, 2,6-ArH), 8.22 (2H, d, *J* = 8.70, 3,5-(ArH)), and 9.9 (1H, s., CHO). ^13^C NMR (CDCl_3_, δ, ppm): 191.00, 163.81, 136.26, 132.08, 129.92, 126.54, 114.84, 67.94, 45.43, 33.19, 28.46, and 25.14.

#### 2.1.2. Synthesis of 5-(4-Acetamidophenyl)-10,15,20-tris(4-(4-bromo-n-butoxy)phenyl)porphyrin **2**

In 200 mL of CH_2_Cl_2_ pyrrole (0.20 g, 3.00 mmol), 4-(4-bromo-n-butoxy)benzaldehyde (0.580 g, 2.20 mmol) and 4-acetamidobenzaldehyde (0.122 g, 0.75 mmol) were dissolved. The reaction mixture was stirred under an argon atmosphere and then 40 µL of boron trifluoride etherate was added (0.28 mmol). DDQ (0.681 g, 3.00 mmol) was added for 40 min. The reaction mixture was extracted in a CH_2_Cl_2_/H_2_O medium, and the organic layer was concentrated in vacuo. The target products were purified using column chromatography on silica gel G60 (CH_2_Cl_2_:Ethyl acetate = 30:1 *v/v*). Yield: 0.174 g (15%). R_f_ = 0.5 (CH_2_Cl_2_:Ethyl acetate = 30:1 *v/v*). EAS (λ_max_, nm, CH_2_Cl_2_, (lgε)): 423.4 (5.70); 519.4 (4.35), 558.8 (4.10), 594.4 (3.99), and 652.6 (3.88). ^1^H-NMR (CDCl_3_, δ, ppm): −2.75 (2H, s, NH-pyrrole), 2.07–2.17 (6H, m, CH_2_CH_2_Br), 2.19–2.28 (6H, m, OCH_2_CH_2_), 2.30 (3H, s, NHC(O)CH_3_), 3.62 (6H, t, *J* = 6.40 Hz CH_2_Br), 4.18 (6H, t, *J* = 6.50 Hz, OCH_2_), 7.21 (6H, d, *J* = 10.86 Hz 3,5-ArH), 7.81 (2H, d, *J* = 8.20 Hz, 3,5-ArH), 8.03–8.16 (8H, m, 2,6-ArH), and 8.85 (8H, d, *J* = 6.70 Hz, β-CH-pyrrole). ^13^C-NMR (CDCl_3_, δ, ppm): 170.32, 157.23, 147.36, 138.67, 134.87, 133.25, 132.54, 130.85, 129.15, 120.65, 118.23, 116.74, 115.06, 67.58, 34.92, 29.68, 29.16, and 22.45. MALDI-TOF mass spectrum: *m/z*—found 1120.434 [M+1]^+^ (calculated for C_58_H_54_Br_3_N_5_O_4_: 1121.17).

#### 2.1.3. Synthesis of 5-(4-Aminophenyl)-10,15,20-tris(4-(4-bromo-n-butoxy)phenyl)porphyrin **3**

A total of 0.050 g (0.10 mmol) of 5-(4-acetamidophenyl)-10,15,20-tris(4-(4-bromo-*n*-butoxyphenyl))porphyrin was dissolved in 8 mL of trifluoroacetic acid. Then, 12 mL of concentrated hydrochloric acid was added for 30 min. The reaction mixture was stirred and boiled in an oil bath for 40 h at 80 °C; cooled to room temperature; and extracted in a methylene CH_2_Cl_2_/H_2_O system. The resultant solution was washed with aqueous ammonia solution until the organic layer changed color from green to violet. The reaction mixture was concentrated under vacuum. The target product was purified via column chromatography on G 60 silica gel, which was eluted with a CH_2_Cl_2_:Ethyl acetate system (70/1). The product was dried in vacuo over P_2_O_5_. The yield was 0.045 g (87%). R_f_ = 0.6 (CH_2_Cl_2_/ethyl acetate = 70/1). EAS (λ_max_, nm, CH_2_Cl_2_, (lgε)): 423.8 (5.75); 519.9 (4.36), 559.2 (4.05), 594.7 (3.25), and 652.4 (3.06). FTIR: 3376.95–3313.76 (st NH_2_) and 1650–1590 (δ NH_2_). ^1^H-NMR (CDCl_3_, δ, ppm): −2.74 (2H, s, NH-pyrrole), 2.07–2.24 (12H, m, OCH_2_CH_2_CH_2_CH_2_Br), 3.76 (6H, t, *J* = 6.10 Hz, CH_2_Br), 4.28 (6H, t, *J* = 6.50 Hz OCH_2_), 7.05 (2H, d, *J* = 8.40 Hz, 3,5-ArH), 7.25 (6H, d, *J* = 8.50 Hz, 3,5-ArH), 7.99 (2H, d, *J* = 8.30 Hz, 2,6-ArH), 8.11 (6H, d, J = 8.60 Hz, 2,6-ArH), and 8.89 (8H, dd, *J*_1_ = 22.90 Hz, *J*_2_ = 3.90 Hz, β-CH-pyrrole). ^13^C-NMR (CDCl_3_, δ, ppm): 156.93, 146.12, 145.05, 134.71, 133.86, 132.61, 131.04, 128.96, 120.29, 114.97, 113.64, 110.90, 66.12, 35.42, 29.12, and 28.88. MALDI-TOF mass spectrum, *m/z*: found 1077.95 [M+1]^+^ (calculated for C_56_H_52_Br_3_N_5_O_3_: 1079.16).

#### 2.1.4. Synthesis of 5-(4-Azidophenyl)-10,15,20-Tris(4-(4-bromo-n-butoxy)phenyl)porphyrin **4**

In a round bottom flask, 0.112 g (0.103 mmol) of porphyrin **3** was dissolved in 10 mL of TFA. A solution of 0.014 g (0.21 mmol) of sodium nitrite in 5 mL of water was added to the resulting solution for 15 min. The resulting mixture was left to stir in an ice bath at 0 °C for 30 min, after which a solution of 0.027 g (0.42 mmol) of sodium azide in 5 mL of water was added to the reaction mixture for 15 min. The second stage of the reaction was performed at room temperature. After completion of the reaction, the mixture was extracted in a methylene-chloride/water system with an aqueous ammonia solution until the organic layer changed color from green to burgundy. The organic layer was evaporated using a rotary evaporator. To isolate the target compound, the reaction mixture was column-chromatographed on G 60 silica gel. The target compound was eluted in an organic solvent system, namely, methylene chloride/hexane = 3/1. The target compound was eluted with the first fraction. It was dried in vacuo over P_2_O_5_. The yield was 0.091 g (80%). R_f_ = 0.85 (CH_2_Cl_2_). IR spectrum (ν, cm^−1^): 3020 (C-H); 2950 (ν-CH_3_); 2150–2200(-N_3_); 1450–1580 (NH, pyrrole); 1300 (C-O); 1150–1290 (-N_3_); 1020 (Ar-O-); 860 (1,4- ArH); 820 (δ-CH-Ar); and 750 (δ-(CH_2_)_4_). EAS (λ_max_, nm, CH_2_Cl_2_, (lgε)): 423.6 (5.76); 519.6 (4.35), 558.0 (4.04), 594.4 (3.25), and 652.5 (3.07). ^1^H-NMR (CDCl3, δ, ppm): −2.74 (2H, c, NH-pyrrole), 2.04–2.20 (12H, m, OCH_2_CH_2_CH_2_CH_2_Br), 3.75 (6H, t, *J* = 6.00 Hz, CH2Br), 4.23 (6H, t, *J* = 6.10 Hz, OCH_2_), 7.21 (6H, d, *J* = 7.60 Hz, 3,5-ArH), 7.32 (2H, d, *J* = 7.20 Hz, 3,5-ArH), 8.10 (6H, d, *J* = 6.80 Hz, 2,6-ArH) 8.18 (2H, d, *J* = 7.20 Hz, 2,6-ArH), and 8.91–8.98 (8H, m, β-CH-pyrrole). ^13^C-NMR (CDCl_3_ δ, ppm): 158.91, 151.66, 151.00, 148.08, 142.75, 141.53, 134.85, 133.29, 132.13, 131.89, 131.46, 130.73, 126.68, 120.65, 120.00, 116.97, 114.08, 69.64, 33.04, 29.20, and 28.93.

#### 2.1.5. Synthesis of Zn (II) Complex of 5-(4-Azidophenyl)-10,15,20-tris(4-(4-bromo-n-butoxy)phenyl)porphyrin **5**

A solution of 0.062 g (0.249 mmol) of zinc acetate dihydrate in 5 mL of methanol was added to a solution of 0.030 g (0.023 mmol) of porphyrin **4** in 10 mL of methylene chloride; then, the mixture was stirred for 3 h. The mixture was extracted in a dichloromethane/water system. The organic layer was collected and evaporated on a rotary evaporator. The purple solid was purified by column chromatography on G60 silica gel using the following solvent system: CH_2_Cl_2_/hexane = 8/1. The target compound was eluted with the first fraction. The product was recrystallized from methanol. It was dried in vacuo over P_2_O_5_. The yield was 0.030 g (94%). R_f_ = 0.82 (CH_2_Cl_2_). IR spectrum (ν, cm^−1^): 3020 (C-H); 2950 (ν-CH_3_); 2150–2200(-N_3_); 1450–1580 (NH, pyrrole); 1300 (C-O); 1150–1290 (-N_3_); 1020 (Ar-O-); 860 (1,4- ArH); 820 (δ-CH-Ar); and 750 (δ-(CH_2_)_4_). EAS (λ_max_, nm, CH_2_Cl_2_, (lgε)): 424.0 (6.17), 558.3 (4.69), 594.8 (4.22). ^1^H-NMR (CDCl_3_, δ, ppm): 2.08–2.18 (12H, m, OCH_2_CH_2_CH_2_CH_2_Br), 3.69 (6H, t, *J* = 5.90 Hz, CH2Br), 4.43 (6H, t, *J* = 6.00 Hz, OCH2), 7.26 (6H, d, *J* = 7.80 Hz, 3,5-(ArH)), 7.35 (2H, d, *J* = 7.20 Hz, β-ArN3), 8.15 (6H, d, *J* = 7.00 Hz, 2,6-(ArH)) 8.19 (2H, d, *J* = 7.40 Hz, α –ArN3), and 8.94–8.99 (8H, m, β-CH-pyrrole). MALDI-TOF mass spectrum, *m*/*z*: found 1171.06 [M]+ (calculated for C57H51Br3N7O3Zn: 1171.06).

#### 2.1.6. Synthesis of Erlotinib-Porphyrin Conjugate **6**

A total of 0.0011 g (0.0073 mmol) of erlotinib was added to a solution of 0.030 g (0.022 mmol) of compound **5** in 8 mL of THF. A solution of 0.010 g of copper (II) sulfate pentahydrate and 0.015 g of sodium ascorbate solution in 1 mL of water was added to the resulting mixture. The reaction mixture was boiled for 15 h. Then, the reaction mixture was extracted in THF/saturated NaCl solution. The collected organic layer was dried with sodium sulfate and evaporated using a rotary evaporator. The purple solid was purified by column chromatography on G60 silica gel. Methylene-chloride/methanol solvent mixture was used as an eluent in a 30/1 volume ratio. The target compound was eluted with the second fraction. The solvent was evaporated using a rotary evaporator. It was then dried in vacuo over P_2_O_5_. The yield was 0.015 g (68%). Rf = 0.4 (CH_2_Cl_2_/CH_3_OH=30/1). R_f_ = 0.4 (CH_2_Cl_2_/CH_3_OH=30/1). EAS (λ_max_, nm, THF, (lgε)): 424.5 (5.79), 558.9 (4.26), and 594.9 (4.06). ^1^H-NMR ((CD_3_)_2_SO, δ, ppm): 2.06 (12H, m, OCH_2_CH_2_CH_2_CH_2_Br), 3.35 (6H, s, OCH_2_CH_2_OCH_3_), 3.79–3.87 (10H, m, OCH_2_CH_2_CH_2_CH2Br and OCH_2_CH_2_OCH_3_), 4.25–4.39 (10H, m, OCH_2_CH_2_CH_2_CH_2_Br and OCH_2_CH_2_OCH_3_), 7.10 (4H, m, 8-H-quinazoline and 3 × Ph-H), 7.27 (2H, s, 3,5-ArH), 7.36 (6H, d, J = 8.20 Hz, 3,5-ArH), 7.59 (1H, m, 5-H-quinazoline), 7.80 (1H, t, *J* = 6.16 Hz, Ph-H), 8.00 (2H, d, *J* = 7.20 Hz, 2,6-ArH), 8.09 (6H, d, *J* = 7.40 Hz, 2,6-ArH), 8.52 (1H, s, pyrimidine-H), 8.55 (1H, s, triazole-H), and 8.84 (8H, m, β-CH-pyrrole). Mass spectrum, *m/z*: found 1596.971 [M+Na]^+^ (calculated for C_78_H_71_Br_3_N_10_O_7_Zn 1565.23).

#### 2.1.7. Synthesis of Erlotinib-Porphyrin Conjugate **7**

A total of 0.033 g (0.021 mmol) of compound **6** was dissolved in 15 mL of anhydrous pyridine. The reaction mixture was boiled under reflux for 3 h. The precipitate from the synthesis was filtered off and washed with methylene chloride. The reaction mixture, which was dissolved in a non-polar solvent, was concentrated under vacuum. Then, it was dissolved in methanol and recrystallized using medical ether. The target compound was dried under reduced pressure over P_2_O_5_. The yield: 0.0225 g (93%). R_f_ = 0.30 (CH_3_OH). EAS (λ_max_, nm, MeOH, (lgε)): 424.3 (6.16), 557.9 (4.66), and 593.9 (4.18). ^1^H-NMR (CD_3_OD, δ, ppm): 1.91–2.11 (12H, m, OCH_2_CH_2_CH_2_CH_2_Py), 3.35 (6H, s, OCH_2_CH_2_OCH_3_), 3.88 (10H, m, OCH_2_CH_2_CH_2_CH_2_Py and OCH_2_CH_2_OCH_3_), 4.37 (10H, m, OCH_2_CH_2_CH_2_CH_2_Py and OCH_2_CH_2_OCH_3_), 7.08 (2H, d, *J* = 8.13 Hz, 3,5-ArH), 7.20 6H, d, *J* = 8.05 Hz, 3,5-ArH), 7.40 (1H, s, Ph-H), 7.52 (1H, t, *J* = 5.68 Hz, Ph-H), 7.71 (2H, m, Ph-H), 7.82 (2H, d, *J* = 8.18 Hz, 2,6-ArH), 7.95 (6H, d, *J* = 8.13 Hz, 2,6-ArH), 8.21 (8H, m, 3,5-Py and 5,8-H-quinazoline), 8.60 (1H, s, pyrimidine-H), 8.89 (12H, m, 4-Py and β-CH-pyrrole and triazole-H), and 9.13 (6H, d, 7.20 Hz, 2,6-Py). Mass spectrum, *m*/*z*: found 520.866 [M]^3+^ (calculated for C93H82N13O7Zn 1556.57).

### 2.2. Aggregation Behavior, Photophysical Characterization, and Solubilization Studies

In the solubilization studies, 10 μL of a stock porphyrin solution in DMSO (CM = 0.3–0.5 mM) was added and stirred into 3 mL of an aqueous solubilizer solution. Porphyrin concentration in the final solutions was 1–2 μM. Poly-N-vinylpyrrolidone (PVP, Mw = 10,000, Sigma-Aldrich, St. Louis, MI, USA), Cremophor EL^®^ (Cremophor, Sigma Aldrich), Pluronic F-127 (Pluronic, Sigma Aldrich), and bovine serum albumin (BSA, Sigma Aldrich) were used as solubilizers. Fluorescence quantum yields (Φ*_F_*) in DMSO were calculated according to a standard procedure [36,37] using ZnTPP (Φ*_F_* = 0.033) as a standard [38,39,40]. The error of the fluorescence quantum yield estimation was 10%.

### 2.3. Photochemical Study

For the photochemical studies, the samples were irradiated in 10 mm quartz cells at room temperature in air-saturated solutions. The light sources included a 150 W halogen lamp, a three-lens spherical condenser with a reflector, and thermal and UV filters. The incident light intensity was 10 mW/cm^2^. Photostability of the porphyrins dissolved in DMSO and in aqueous solubilizer solutions was estimated according to the absorbance decrease in the Soret band upon the sample’s irradiation with white light using the light sources described above without any Vis cut-off filters. For the determination of the singlet oxygen quantum yield, a 500 nm cut-off filter was added to the optical scheme described above. The photosensitized singlet oxygen generation efficiency of the porphyrin solutions was estimated from the absorbance decrease at 418 nm, corresponding to the concentration of the selective ^1^O_2_ acceptor—1,3-diphenylisobenzofuran (DPBF), which was added to the porphyrin solution in DMSO immediately before the start of irradiation (C_DPBF_ = 0.1 mM). In all the photochemical experiments, porphyrin concentration was maintained at 1–2 μM to minimize the internal filter effect. Calculation of the singlet oxygen quantum yield (Φ_Δ_) was carried out according to a procedure described elsewhere [41,42], for which ZnTPP was used as a standard (Φ_Δ_ = 0.74) [43]. The error of the determination of the singlet oxygen quantum yield was 10%.

### 2.4. 1-n-Octanol/PBS Partition Coefficients

The partition coefficients of the studied compounds were determined according to a previously described method [44]. Briefly, 1.0–1.5 mg of porphyrins was dissolved in 1-*n*-octanol solution that was pre-saturated with phosphate-buffered saline (PBS). The same volume of PBS saturated with 1-*n*-octanol was added. The reaction mixture was sonicated for 30 min and agitated at 25 °C. Phases were separated via centrifugation. Partition coefficients were calculated via electronic absorption spectroscopy as the ratio between absorption in the Soret band of porphyrins in organic and aqueous phases and the dilution factors for the organic and aqueous layers determined according to the same method. Values are presented as LogP.

### 2.5. Encapsulation of Porphyrins into Pluronic Micelles

Porphyrins were encapsulated into polymer micelles according to the following method: for 30 min, a solution of porphyrin (1 mg/mL) in methanol (15 mL) was added to a solution of Pluronic F127 with a concentration of 15 mg/mL in methanol (15 mL). The resulting mixture was stirred vigorously and evaporated using a rotary evaporator. The product was dried in dynamic vacuum. The hydrodynamic radii (Rh) of the received nanoparticles were determined using dynamic light scattering (DLS) by employing Delsa Nano (Beckman Coulter, Inc., Brea, CA, USA).

### 2.6. Cell Culture

The cancer cell line MCF-7 (human breast adenocarcinoma) was purchased from the American Type Culture Collection (ATCC, Manassas, VA, USA). The cancer cell lines NKE (human renal tubule epithelium) and MDA-MB-231 (human breast adenocarcinoma) were purchased from the cell bank of N. N. Blokhin National Medical Research Center of Oncology of the Russian Ministry of Health. MDA-MB-231, NKE, and MCF-7 cells were cultivated in DMEM substratum together with the addition of gentamycin (50 μg/mL) and 10% fetal bovine serum. Cells were cultivated in plastic cell culture flasks (25 cm^2^) at 37 °C in a humidified environment with 5% CO_2_. Cells were seeded before reaching 80% fusion using EDTA/trypsin solution.

For the in vitro experiments, 3-(4,5-dimethyl-2-thiazolyl)-2,5-diphenyl-2H-tetrasolium bromide (MTT) and Mowiol (Sigma–Aldrich); 96% ethanol (Chimmed, Moscow, Russia); fetal bovine serum (FBS) (Gibco, Waltham, MA, USA); Dulbecco’s modified Eagle’s medium (DMEM) (Gibco); dimethyl sulfoxide (DMSO) (Amreso, Solon, OH, USA); 0.02% EDTA and 0.05% trypsin solutions (Gibco); and gentamycin (PanEco, Moscow, Russia) were used. All chemicals were used without further purification. Milli-Q water was used in the experiments.

### 2.7. In Vitro Internalization Assay

Fluorescence intensity of the photosensitizers in MCF-7 cells was analyzed using flow cytometry. MCF-7 cells were seeded in 6-well plates at a density of 2 × 10^5^ cells per well and allowed to adhere overnight. The cells were treated with 3 μM of porphyrins for 2 h, 4 h, and 24 h. Then, cells were collected using EDTA/trypsin solution, washed twice with saline phosphate buffer, and analyzed. The intensity of fluorescence was registered as the average value of fluorescence intensity (MFI) using a Cyan ADP (Beckman Coulter, Inc.) flow cytometer equipped with 665/20 nm bandpass filter and λex 635 nm laser.

### 2.8. In Vitro Cell Viability (MTT Test)

This experiment was performed using two cell lines: NKE (human renal tubule epithelium) and MDA-MB-231 (human breast adenocarcinoma). In the experiment, cells were seeded into 96-well plates (SPL Lifesciences, Pocheon-si, Korea) at 7 × 10^3^ cells/well in 180 μL of culture medium and incubated for 24 h at 37 °C in 5% CO_2_. On the day of the experiment, the medium was replaced with a similar one but which did not contain serum to avoid its interaction with the studied compounds. Serial dilutions of the compound were created in deionized water and added to the cell culture by 20 μL each. The cell culture was irradiated for 90 min using a Medical Therapy Philips TL 20W/52 lamp (wavelength 400–500 nM) with 2.3 mW of power. Subsequently, the plates were placed in an incubator and left for 24 h at 37 °C in 5% CO_2_. Parallel incubation of cell culture with compounds without irradiation was performed in a similar manner. After 24 h, 10 μL of MTT reagent solution was added to the cell medium and incubated for another 3.5 h. The formazan forming in the cells was dissolved in DMSO (100 μL). MultiScan MCC 340 spectrophotometer (Labsystems, Boston, MA, USA) was used to determine the optical density of the solution at 540 nm. Experiments were repeated at least 3 times. The titration curves were used to calculate the concentration of the compound that induced 50% of the maximum toxicity effect (IC_50_). The Excel program package (Microsoft Corporation, Redmond, WA, USA) was utilized for statistical processing of the obtained data.

### 2.9. Statistical Analysis

The Excel (Microsoft Corporation) and OriginPro 8.0 (OriginLab Corporation, Northampton, MA, USA) program packages were used for data visualization and statistical analysis. The mean ± standard deviation was calculated, and one-way analysis of variance was utilized to determine the significant difference. Outcomes for which *p* < 0.05 were considered statistically significant.

## 3. Results

### 3.1. Synthesis

**Scheme 1:**
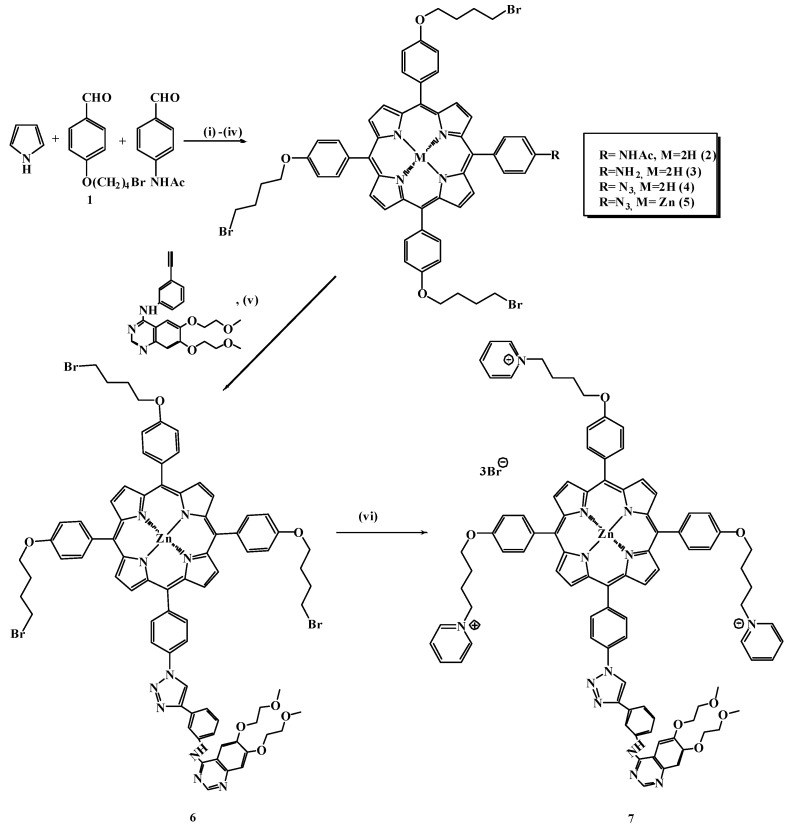
Reagents and reaction conditions: (i) CHCl_3_, BF_3_·Et_2_O, DDQ, and rt; (ii) TFA, HCl, and 80 °C; (iii) TFA, NaNO_2_, and 0 °C, and then the addition of NaN_3_ at rt; (iv) ZnOAc_2_ and CH_2_Cl_2_/CH_3_OH; (v) Erlotinib, sodium ascorbate, CuSO_4_·5H_2_O, THF/H_2_O, and reflux; (vi) pyridine and reflux.

In the first stage of this study, we developed a method for the preparation of cationic porphyrins of the A3B type containing a ω-bromalkyl spacer and a reactive amino group (Scheme 1). Benzaldehyde **1** was obtained using the standard method entailing the O-alkylation of 4-hydroxybenzaldehyde with 1,4-dibromobutane in acetone in the presence of excess potassium carbonate base (5 eq.) at a 76% yield. The synthesis of porphyrin was carried out using a mixed-aldehyde monopyrrole condensation reaction according to Lindsey’s method [45]. It was found that carrying out this reaction under the conditions stipulated by a modified version of Adler’s method [46,47] (boiling in a mixture of propionic and acetic acids and nitrobenzene) leads to the formation of a large number of elimination products from the terminal methylene group. The Lindsey condensation reaction proceeded in mild conditions: at room temperature in chloroform using a Lewis catalyst, namely, boron trifluoride etherate (BF_3_·Et_2_O). Then, the acetyl protecting group was removed via boiling and stirring in trifluoroacetic acid (TFA) with a gradual introduction of concentrated hydrochloric acid drop by drop. The yield of compound **3** was 87%, and its structure and identity were confirmed by TLC, ^1^H-NMR, IR, and UV spectroscopy as well as MALDI-TOF mass spectrometry. In the ^1^H NMR spectrum of compound **3**, signals from β- pyrrole protons were observed at 8.85–8.91 ppm, and doublet signals from protons of phenyl groups at 7.85 and 7.25 ppm with an integral intensity of 1:3 were also observed, indicating the presence of an asymmetric, A3B-type system of the porphyrin (Figure S6, Supplementary Materials). Compared with porphyrin **2**, the disappearance of signals from the acetyl-group protons in the strong field region was recorded, confirming the exhaustive removal of the acetyl protection. In the IR spectrum of compound **3** in the regions of 3380–3310 cm^−1^ and 1650–1590 cm^−1^, there were signals from the valence and strain vibrations of the primary amino group.

The azide–alkyne cycloaddition reaction with the formation of 1,2,3-triazoles via the Huisgen–Meldahl–Charpless method has been widely used in the chemistry of porphyrins to obtain various conjugates [48,49]. The triazole spacer functions as a rigid binding “bridge” that mimics the atomic arrangement and electronic properties of the peptide bond, eliminating the propensity for hydrolytic cleavage [50,51]. In the first step of our work, an azide-containing component based on aminoporphyrin **3** was prepared via the action of a mixture of TFA and aqueous sodium nitrite solution at 0 °C to avoid side products and strong exothermic effects. The resulting diazo compound was treated with an aqueous sodium azide solution at room temperature (Scheme 1) to obtain the target product **4**. Symmetric and asymmetric valence vibrations of the azide group in the region of 2150–2200 cm^−1^ and 1150–1290 cm^−1^ were observed in the IR spectra (Figure S10, Supplementary Materials). In the ^13^C-NMR spectrum of azide **4**, a peak at 70 ppm corresponding to the signal of the phenyl group bound to the azide was observed. Further, a complex of porphyrin **4** with Zn(II) was obtained since copper salts capable of complexing with the initial porphyrin participate in the click reaction. The formation of a complex with Zn(II), where four Q-bands degenerated to two due to the increased symmetry of the macrocycle, was confirmed by electronic absorption spectra.

**Figure 1:**
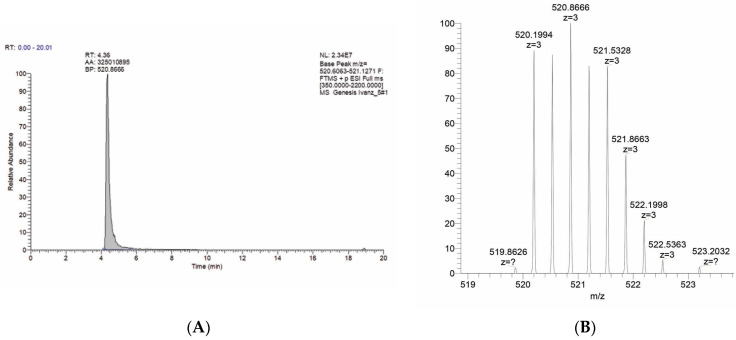
(**A**) Mass chromatogram of target compound **7**; the retention time of the target compound is 4.38 min. (**B**) Fragment of mass spectrum of **7** *m*/*z* [M+H]^3+^ = 520.8666.

The key step in the synthesis of porphyrin with the targeting ligand **6** consisted of the 1,3-bipolar cycloaddition of erlotinib with a terminal triple bond to azidoporphyrin. Commercially available erlotinib was used as the acetylene component, which was treated with zinc azidoporphyrin complex **5**. An aqueous mixture of copper sulfate and sodium ascorbate, which generates a salt of univalent copper in situ, served as a catalyst for the reaction. The optimal conditions for this reaction were determined. Accordingly, the reaction mixture was boiled in a THF/H_2_O mixture for 15 h. The target product was isolated chromatographically; the yield of compound **6** was 68%, indicating the high efficiency of the synthesis. The final stage of synthesis was to obtain the cationic erlotinib–porphyrin conjugate **7** via the pyridine quaternization reaction. Compound **6** was boiled in pyridine for 3 h, with the target conjugate precipitating during the reaction. The structures and identities of all the obtained compounds were confirmed via TLC, ^1^H-NMR, ^13^C-NMR, and UV spectroscopy as well as MALDI-TOF mass spectrometry; HPLC HRMS analysis was performed for the target conjugate (Figure 1A,B and Supplementary Figures S1–S18).

### 3.2. Aggregation Behavior, Photophysical Characterization, and Solubilization Studies

**Figure 2:**
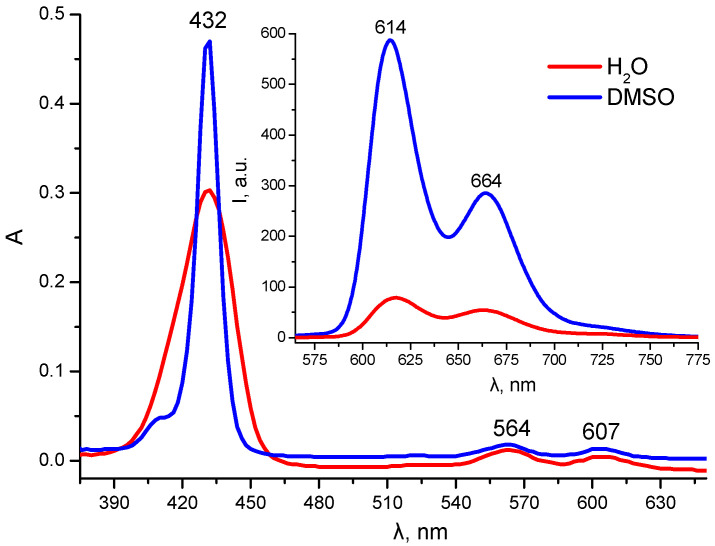
UV–Vis absorption and fluorescence (insert) spectra of 7 (C = 1.15 μM) in DMSO and H_2_O (λ_ex_ = 432 nm).

The UV–Vis absorption spectrum of compound **7** in DMSO is rather typical of metal porphyrin complexes, with a Soret band maximum at 432 nm (lgε = 5.69) and two Q-bands at 563 and 603 nm (Figure 2). The corresponding fluorescence spectrum of **7** includes two main emission bands at 614 and 664 nm with a total fluorescence quantum yield of 4.2% (Figure 2, in the insert). A comparison of the absorption and fluorescence spectra of **7** in DMSO and in pure water revealed reduced fluorescence intensity and increased FWHM (from 11 to 28 nm) in aqueous media resulting from porphyrin’s aggregation (Figure 2 and Figure S18). Despite the relatively good water solubility of cationic porphyrin and the obtained negative 1-*n*-octanol/water partition coefficient (Log*Pow* = −1.86), the DLS data indicate the presence of molecular aggregates of several hundred nanometers in diameter in the aqueous solutions of **7** both in distilled water and in phosphate-buffered saline. Thus, the further development of conjugate **7** as a potential photosensitizer requires solubilization studies with the objective of choosing the most appropriate dosage for drug administration.

**Figure 3:**
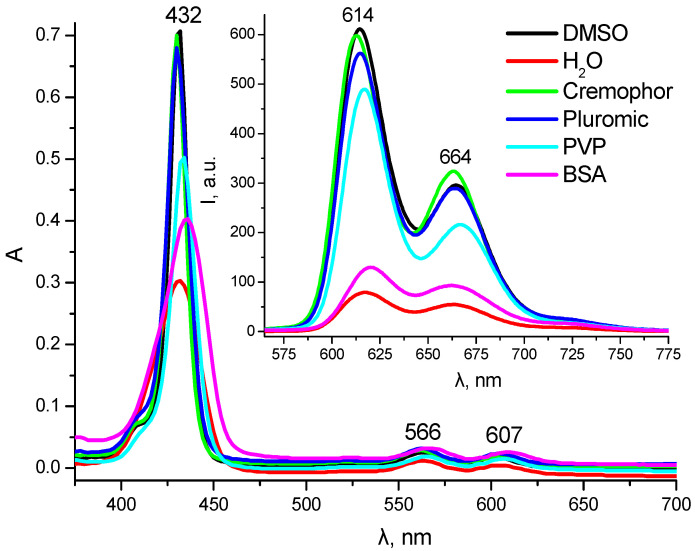
UV–Vis absorption and fluorescence (insert) spectra of **7** (C = 1.8 μM) in DMSO, water, and aqueous solutions of different solubilizers (λex = 432 nm).

In the solubilization studies, the main parameters of the absorption and emission spectra of **7** were compared in aqueous polymer (PVP, BSA) and surfactant (Pluronic, Cremophor) solutions. The data obtained indicate that the most effective stabilization of the monomolecular, fluorescent form of **7** occurred in Cremophor and Pluronic micelles (in both cases, the minimum half-width of the Soret band and the maximum intensity of both absorption and emission bands were observed) and reveal its partial binding with PVP (Figure 3). Upon BSA binding, only a slight fluorescence intensity increase was observed. The above results suggest that Cremophor and Pluronic micellar solutions can be considered the most suitable solubilizers for the photosensitizer studied.

**Figure 4:**
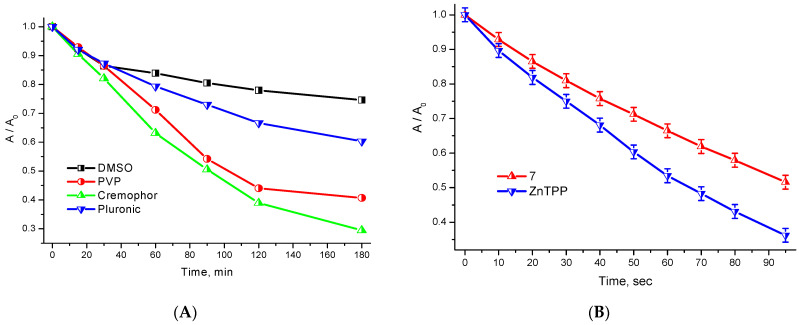
(**A**) Kinetic curves of photobleaching under white light irradiation with respect to **7** in the presence of various solubilizers; (**B**) Kinetic curves of photosensitized DPBF oxidation with singlet oxygen in the presence of **7** and ZnTPP in DMSO.

A comparison of the solubilization efficiency of **7** in the polymer and surfactant concentration range from 1% to 0.01% revealed the following optimal values: Pluronic—1%; PVP—0.25%; and Cremophor—0.1%. The above solubilizer concentrations were used in further photostability studies (Figure 4A). The results obtained indicate a relatively low level of photostability of compound **7** towards direct white light irradiation. For conjugate **7**, the most significant increase in the photobleaching rate was observed in Cremophor micelles (η60 = 37%), while the minimum photobleaching rate was observed in Pluronic polymer micelles (η60 = 21%), which renders the latter preferable for development of a dosage form of compound **7**. In an aqueous solution of PVP, conjugate **7** demonstrated an intermediate photodegradation rate (η60 = 29%).

A comparison of the photosensitized oxidation rate of a selective ^1^O_2_ acceptor—DPBF—in DMSO in the presence of **7** and zinc tetraphenylporphyrin (Figure 4B) revealed the singlet oxygen quantum yield value for conjugate **7**, namely, 58%. Thus, the covalent binding of the cationic porphyrin to the targeting erlotinib fragment does not significantly affect its photophysical properties or photochemical activity in DMSO. However, it significantly affects the HLB of the metal complex, resulting in decreased solubility and an increased tendency towards aggregation in aqueous media. The solubilization studies have shown that the optimal solubilizer for conjugate **7** is a 1% micellar solution of Pluronic F-127 (which exactly corresponds to the doubled cmc value).

### 3.3. Encapsulation of Conjugate into Micelles

The study of the aggregational behavior and solubilization of the target porphyrin–erlotinib conjugate showed the efficiency of using Pluronic F127 polymeric micelles as a potential delivery system. The encapsulation of conjugate **7** was conducted using the solid dispersion method [52,53]. Solubilization was carried out in alcohol solution (methanol), which is a thermodynamically suitable solvent for amphiphilic copolymers [26]. Conjugate **7** and Pluronic F127 (2.5%, *w*/*v*) were dissolved in methanol with subsequent rotatory evaporation of the solvent. The resulting thin solid film was dried in a dynamic vacuum and then dissolved in aqueous solutions. Before the biological studies were conducted, the micellar formulation of **7** was characterized using dynamic light scattering. As can be seen from Table 1, in distilled water, phosphate-buffered saline (PBS), and a basal medium (DMEM) without a solubilizer, despite the presence of cationic groups at the periphery of the macrocycle, compound **7** is dispersed in the form of irregular molecular aggregates with a rather wide particle size distribution (from 200 to 700 nm). However, in the presence of Pluronic polymer micelles, in addition to the porphyrin aggregates, a distinct fraction of nanoparticles of about 24 nm in size is observed, corresponding to the Pluronic micelles loaded with conjugate **7** (Figure S19, Table S1, Supplementary Materials). According to data from the literature [54], pure Pluronic F-127 micelles are characterized by a hydrodynamic diameter of about 20 nm at room temperature. Therefore, the nanoparticles observed correspond to the micellar-solubilized form of photosensitizer **7**.

### 3.4. Cellular Internalization Study

**Figure 5:**
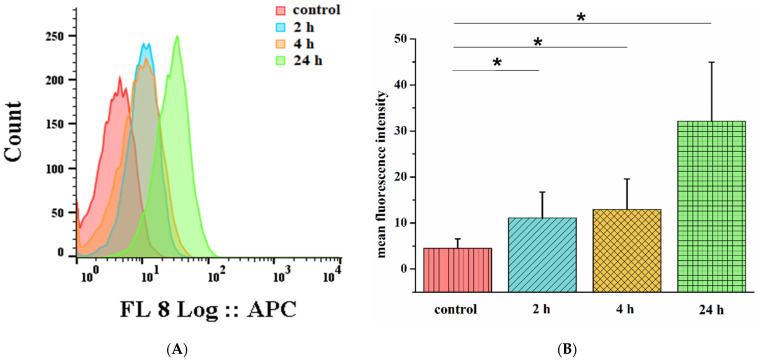
Flow cytometry analysis of MCF-7 cells treated with 3 μM of conjugate **7** for 2 h (blue), 4 h (orange), and 24 h (green), where nontreated control cells are marked in red (**A**). MFI of MCF-7 cells treated with 3 μM of conjugate **7** for 2 h, 4 h, and 24 h (**B**). Data represent mean ± SD and * *p* < 0.05 compared with control.

Due to the detected aggregation of compound **7** in the biological medium (DMEM), a question arose regarding the capacity of cells to internalize this compound. Thus, flow cytofluorometry was applied to evaluate this parameter. We observed a time-dependent increase in conjugate **7**’s fluorescence intensity from 2 h to 24 h after incubation (Figure 5A). However, the fluorescence intensity after 2 h and 4 h of incubation with conjugate **7** was characterized by an insignificant difference. This may be explained by the effect of cell saturation with porphyrins [55,56]. A quantitative fluorescence analysis (Figure 5B) showed the significantly higher fluorescence intensity of conjugate **7** after 24 h of incubation, which may be associated with aggregates and dimers of conjugate **7**’s formation, their accumulation on the cell membrane’s surface, and the involvement of other active endocytosis pathways [57].

As a result of this experiment, it has been determined that despite significant aggregation in a biological medium, compound **7** retains its ability to penetrate cells.

### 3.5. Dark and Photodynamic Activity of PS

The dark and photodynamic activity of conjugate 7 and its Pluronic F127 micellar formulation (**7** + **F127**) was studied in the MDA-MB-231 (human breast adenocarcinoma) and NKE (human renal tubule epithelium) cell lines. Conjugate **7** did not show adequate activity in a biological system. Compound **7** showed no/weak dark toxicity, but cytotoxicity after irradiation was in the range of high concentrations, which are not inherent to this class of compounds. The IC50 values for porphyrins range up to 30 µM [58]. For erlotinib, this value lies at even lower, sometimes nanomolar, concentration ranges [59,60]. The aggregation of compound **7** in an aqueous solution prevents the realization of its biological activity. Thus, despite the demonstrated accumulation of compound **7** in the tested cells, its biological activity appears to be very limited, which is most likely due to its aggregation properties.

**Figure 6:**
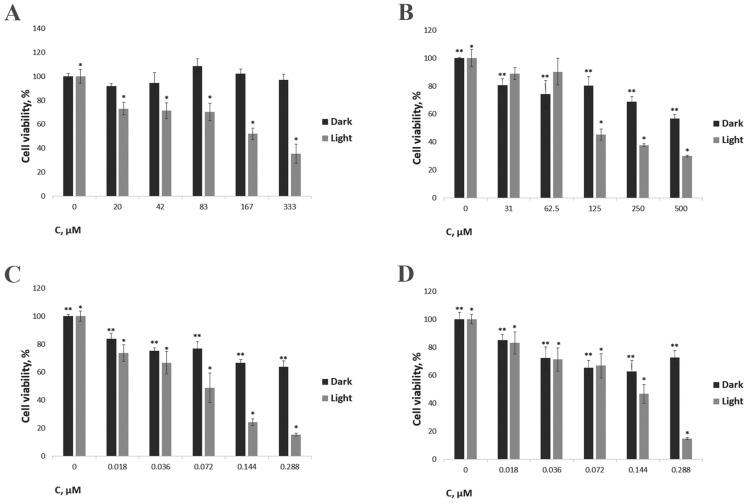
The effect of the developed compound on the viability of MDA-MB-231 and NKE cell lines. (**A**) Conjugate **7** toward MDA-MB-23; (**B**) Conjugate **7** toward NKE; (**C**) Conjugate **7** + F127 toward MDA-MB-23; (**D**) Conjugate **7** + F127 toward NKE. Cells were irradiated for 90 min using a Medical Therapy Philips TL 20W/52 lamp (irradiation dose of 8.073 J/cm^2^). Cells were incubated with the compound for 24 h. */** indicate statistically significant differences in cell survival relative to the values with zero concentration of the compound (*p* < 0.05).

Completely different results were obtained when using the Pluronic F127 micellar formulation (**7** + **F127**). In this case, under dark conditions, the IC_50_ values were 0.99 ± 0.079 μM for MDA-MB-231 and 2.6 ± 0.286 μM for NKE cells, i.e., lying in the range of erlotinib’s toxicity [58]. Moreover, the compound is more toxic toward a tumor cell line characterized by an increased level of EGFR expression, i.e., the MDA-MB-231 cell line, which is also characteristic of erlotinib. After irradiation, the IC_50_ of this compound is 0.073 ± 0.014 μM toward the MDA-MB-231 cell line and 0.13 ± 0.018 μM toward NKE (Table 1, Figure 6). Thus, even a small total irradiation dose (8.073 J/cm^2^) results in a 14–20-fold increase in the compound’s toxicity. At the same time, after irradiation, the compound is 1.8 times more toxic against the EGFR-overexpressing cell line MDA-MB-231 compared to conditionally normal NKE. The obtained results for this new conjugate indicate its relevance and promise within this context and are consistent with the currently known data. Accordingly, in a previously published study concerning a 4-arylaminoquinazoline-containing targeting compound, the light toxicity for EGFR-overexpressing A431 cells was two times higher than the toxicity for weakly expressing EGFR CHO cells [32]. In another study, the compound Hu-CuTPP with an erlotinib delivery system showed 1.3-fold higher toxicity toward the EGFR-overexpressing cell line 4T1 compared to a weakly expressing cell line (LO_2_) [61]. In our work, we obtained similar results, with a significant difference in dark and light toxicity with a total irradiation dose of only 8.073 J/cm^2^.

## 4. Conclusions

In summary, a novel, anticancer, EGRF-targeting photosensitizer based on cationic porphyrin and the small-molecule-targeted drug erlotinib was designed and synthesized. A strategy based on click chemistry proved to be an excellent method for the conjugate’s synthesis. Optimal conditions for reaction and the product’s isolation were established, and all the obtained compounds were characterized by modern physical and chemical methods. In our work, we have shown that the encapsulation of an EGRF-targeted photosensitizer into Pluronic F127 nanomicelles improves its biological properties. Thus, we observed a 14–20-fold increase in photoinduced toxicity compared with the dark. The obtained conjugate presents a 1.8-fold greater toxic effect against the EGFR-overexpressing cell line MDA-MB-231 compared to conditionally normal NKE cells. Our study demonstrates the efficiency of conjugating PS with low-molecular-weight tyrosine kinase inhibitors and using micellar drug carriers as delivery vehicles.

## Figures and Tables

**Table 1:** IC_50_ of compound **7** in the MDA-MB-231 and NKE cell lines.

Cells/Compounds	Conjugate 7 Light *	Conjugate 7 Dark	7 + F127 Light *	7 + F127 Dark
MDA-MB-231	220 ± 20 µM	-	0.073 ± 0.014 µM	0.99 ± 0.079 µM
NKE	150 ± 30 µM	-	0.13 ± 0.018 µM	2.6 ± 0.286 µM

* Dose of irradiation was 8.073 J/cm^2^. Incubation time with the compounds was 24 h.

## Data Availability

The data presented in this study are available in this article and Supplementary Materials.

## Supplementary Materials

The following supporting information can be downloaded at: https://www.mdpi.com/article/10.3390/pharmaceutics15041284/s1. Figures S1–S19, Table S1: spectral data of the synthesized compounds, physicochemical properties, and biological activity.
