# Supplementary material for: Synthesis and Biological Properties of EGFR-Targeted Photosensitizer Based on Cationic Porphyrin
Source: Pharmaceutics. 2023 Apr 19;15(4):1284. doi: 10.3390/pharmaceutics15041284 (PMC10145264; doi:10.3390/pharmaceutics15041284)
Supplement: Supplementary file 1 [file pharmaceutics-15-01284-s001.zip › pharmaceutics-2311296-supplementary.pdf]

Supplementary Materials

Synthesis and Biological Properties of EGRF-targeted Photosensitizer Based on Cationic Porphyrin

Yulia S. Bortnevskaya[a], Nikita A. Shiryaev[a], Nikita S. Zakharov[a], Oleg O. Kitoroage[a], Margarita A. Gradova[b], Natal'ya Yu. Karpechenko[c,d], Alexander S. Novikov[e,f*], Elena D. Nikolskaya[g], Mariia R. Mollaeva[g], Nikita G. Yabbarov[g], Natal'ya A. Bragina[a] and Kseniya A. Zhdanova[a]

[a]MIREA - Russian Technological University, Institute of Fine Chemical Technology, Vernadsky pr., 86, 119571 Moscow, Russian Federation

[b]N.N. Semenov Federal Research Center for Chemical Physics, Russian Academy of Sciences, Kosygin st., 4, 119991 Moscow, Russian Federation

[c]N.N. Blokhin National Medical Research Center of Oncology, Ministry of Health of Russia, Kashirskoe highway, 24, 115522 Moscow, Russian Federation

[d]Pirogov National Research Medical University, Ministry of Health of Russia, Ostrovityanova st., 1, 117997 Moscow, Russian Federation

[e]Institute of Chemistry, Saint Petersburg State University, Universitetskaya nab. 7–9, 199034 Saint Petersburg, Russia

[f]Research Institute of Chemistry, Peoples' Friendship University of Russia (RUDN University), Miklukho-Maklaya st., 6, 117198 Moscow, Russian Federation

[g]Emanuel Institute of Biochemical Physics, Russian Academy of Sciences, Kosygina st, 4, 119334 Moscow, Russia

* Correspondence: a.s.novikov@spbu.ru (A.S.N.)

^{1}H NMR-, ^{13}C NMR, FTIR, mass-spectra of synthesized compounds **1-7** are illustrated below (see **Figures S1–S17**).

4-(4-Bromo-*n*-butoxy)benzaldehyde 1.

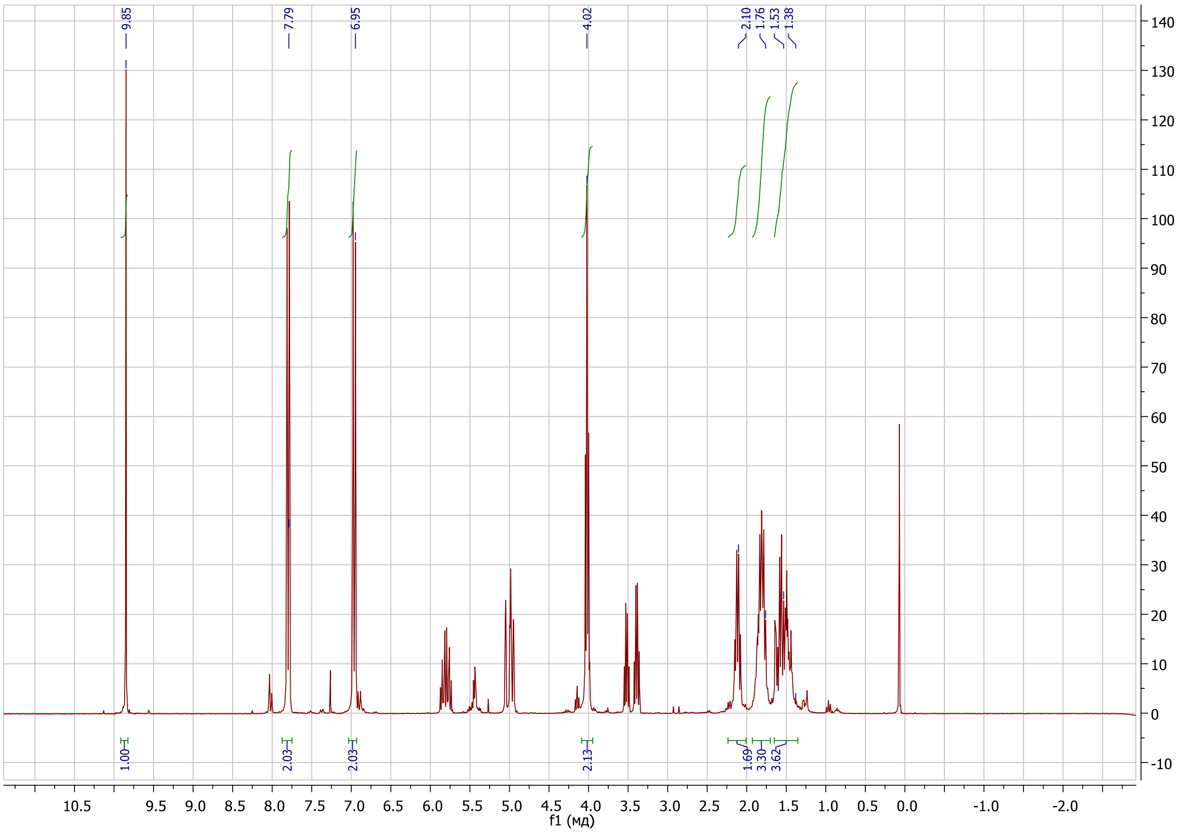

Figure S1. ^{1}H NMR of compound **1** ($CDCl_3$).

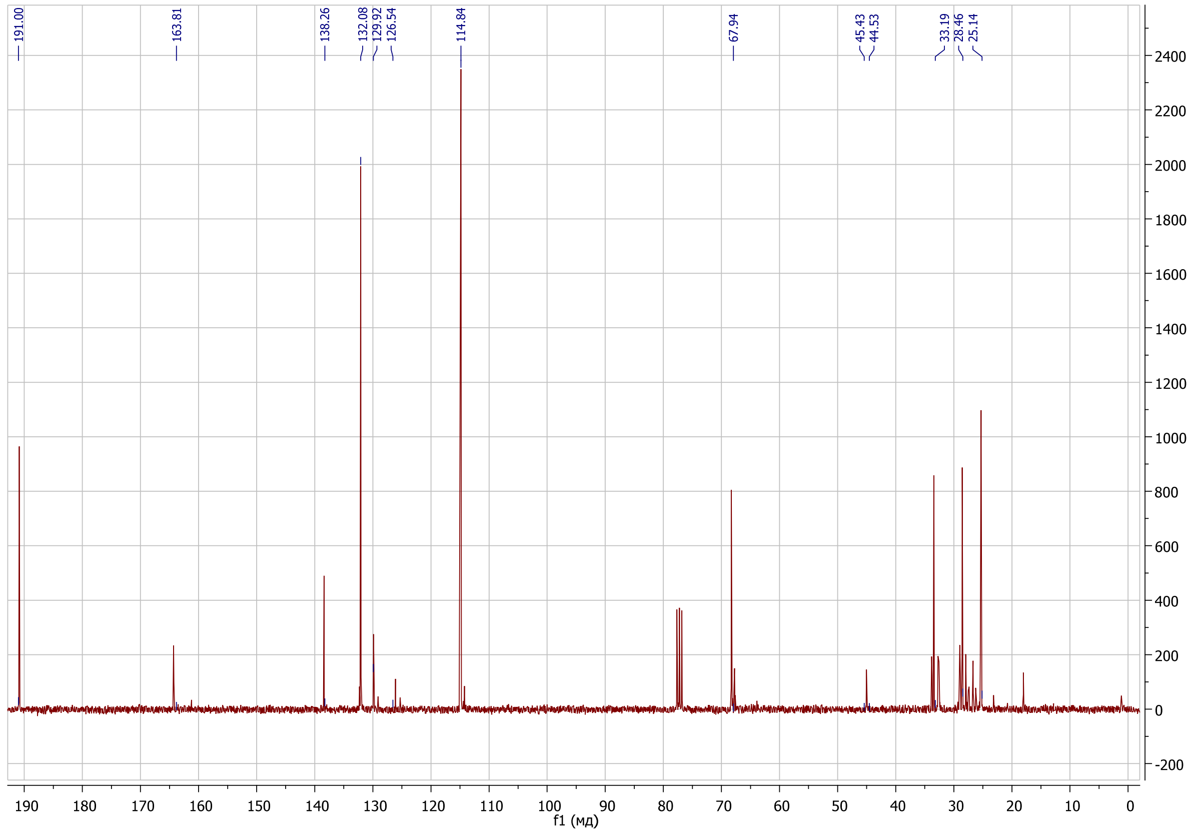

Figure S2. ^{13}C NMR of compound **1** ($CDCl_3$).

5-(4-Acetamidophenyl)-10,15,20-tris(4-(4-bromo-n-butoxy)phenyl)porphyrin 2.

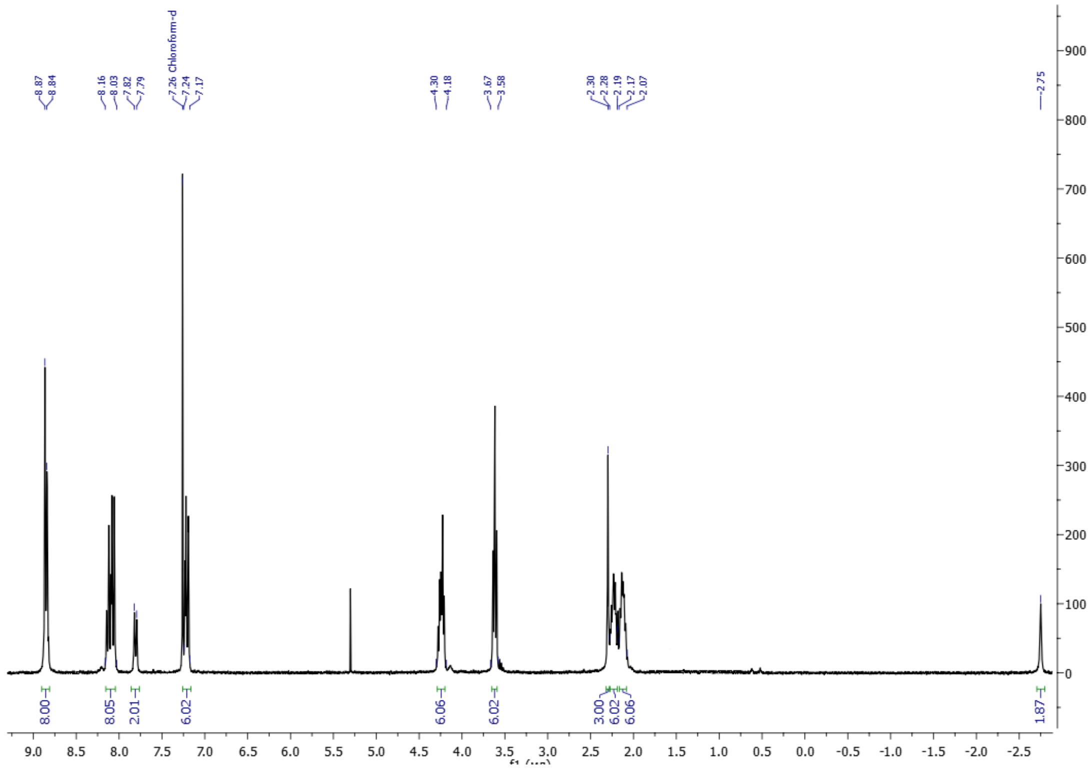

Figure S3. ^{1}H NMR of compound **2** ($CDCl_3$).

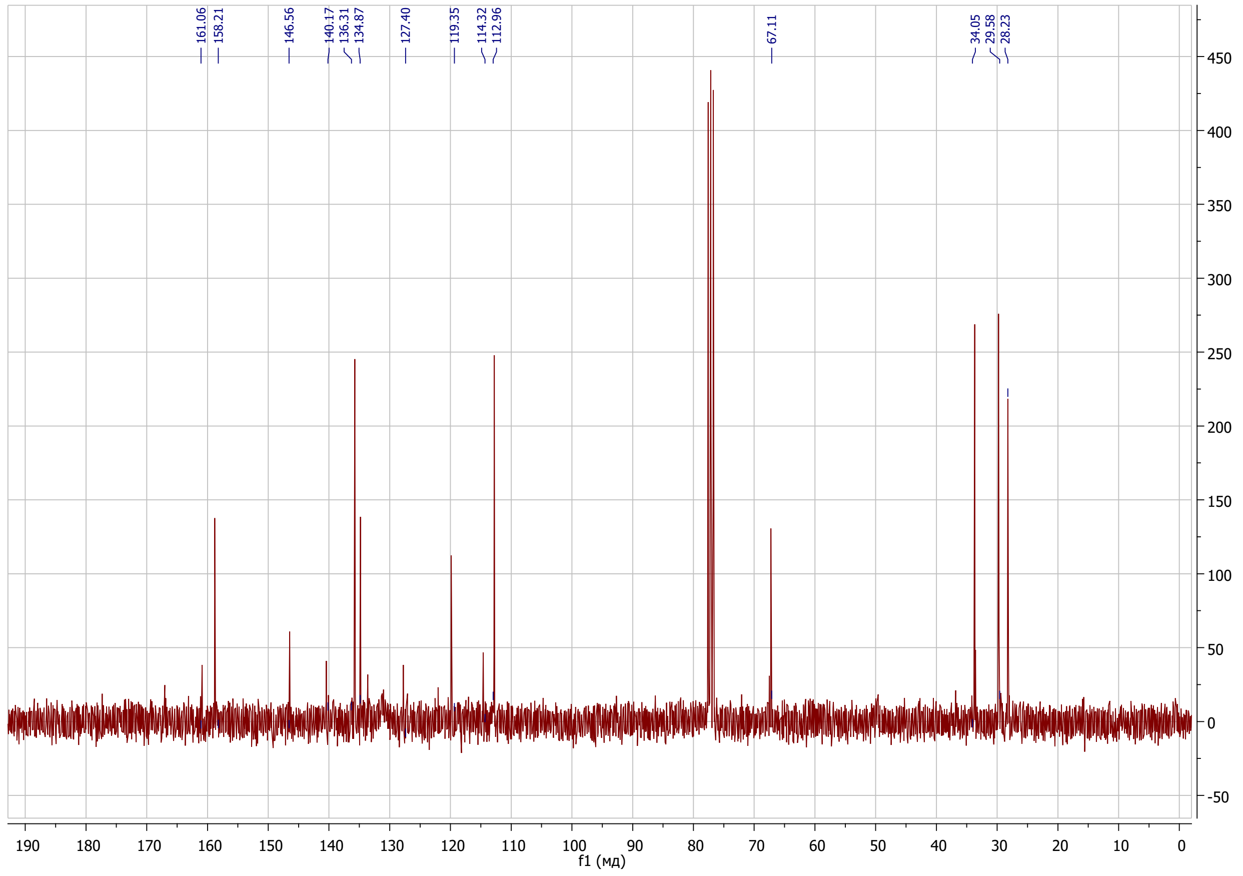

Figure S4. ^{13}C NMR of compound **2** ($CDCl_3$).

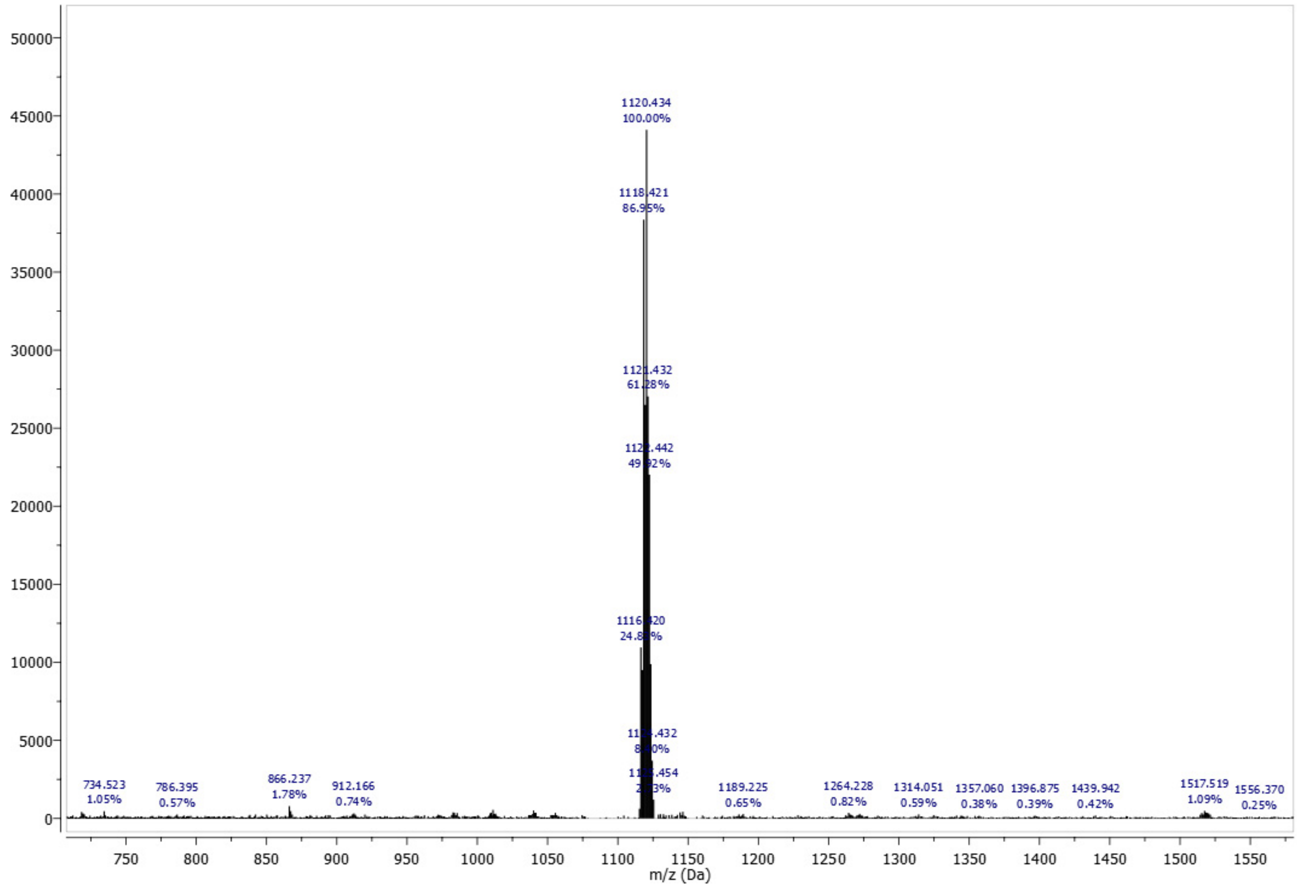

Figure S5. MALDI-TOF mass-spectrum of compound **2**.

5-(4-Aminophenyl)-10,15,20-tris(4-(4-bromo-n-butoxy)phenyl)porphyrin **3**.

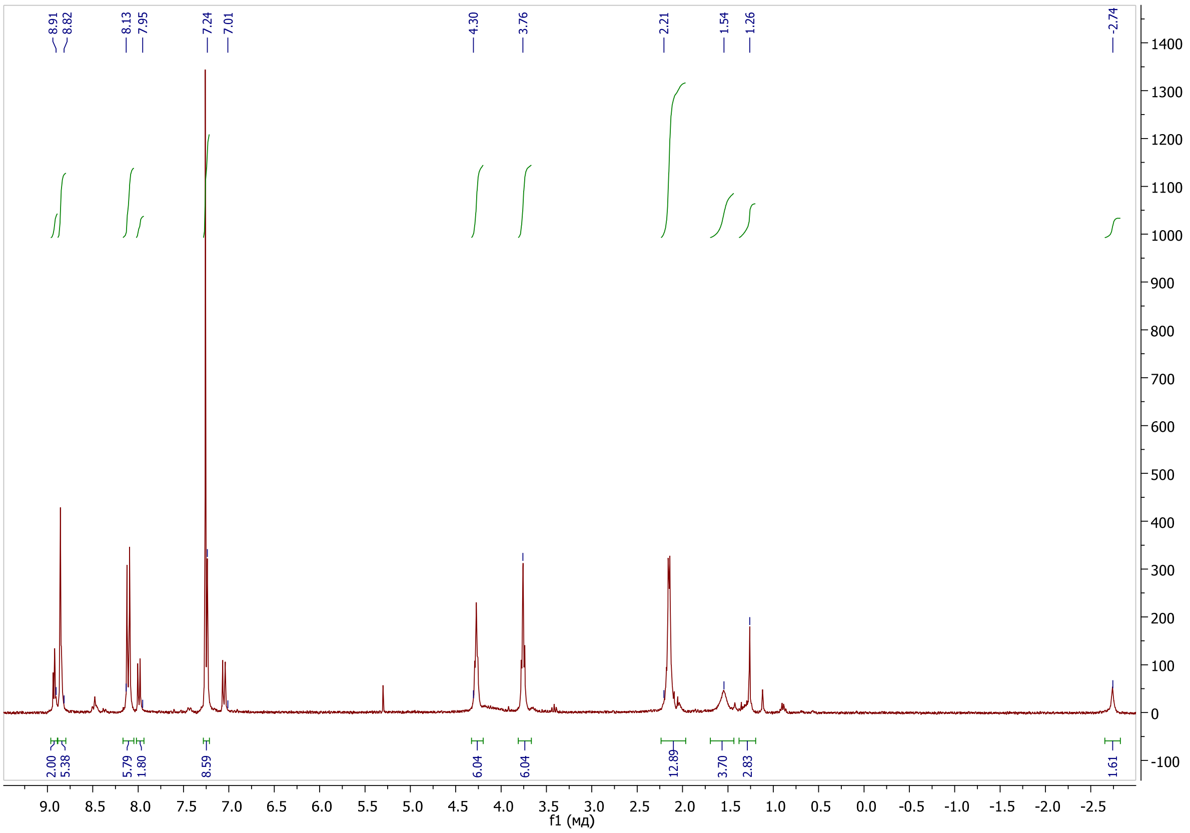

Figure S6. ^{1}H NMR of compound **3** ($CDCl_3$).

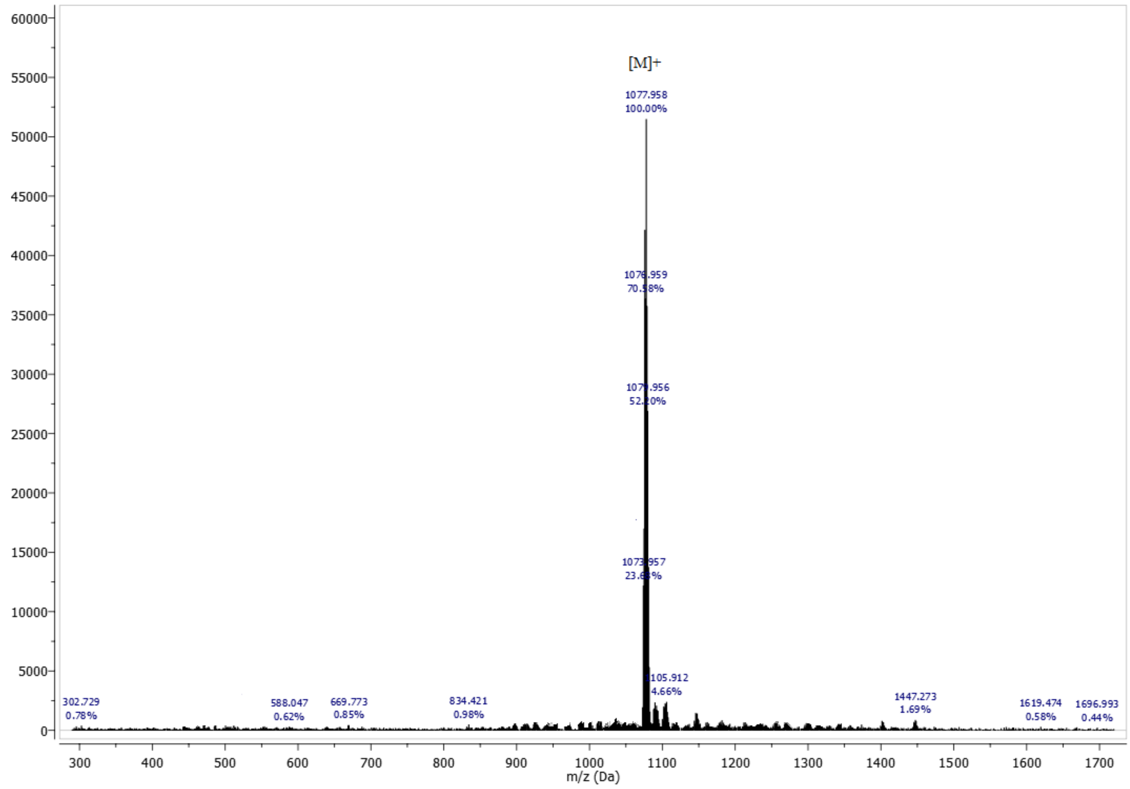

Figure S7. MALDI-TOF mass-spectrum of compound **3**.

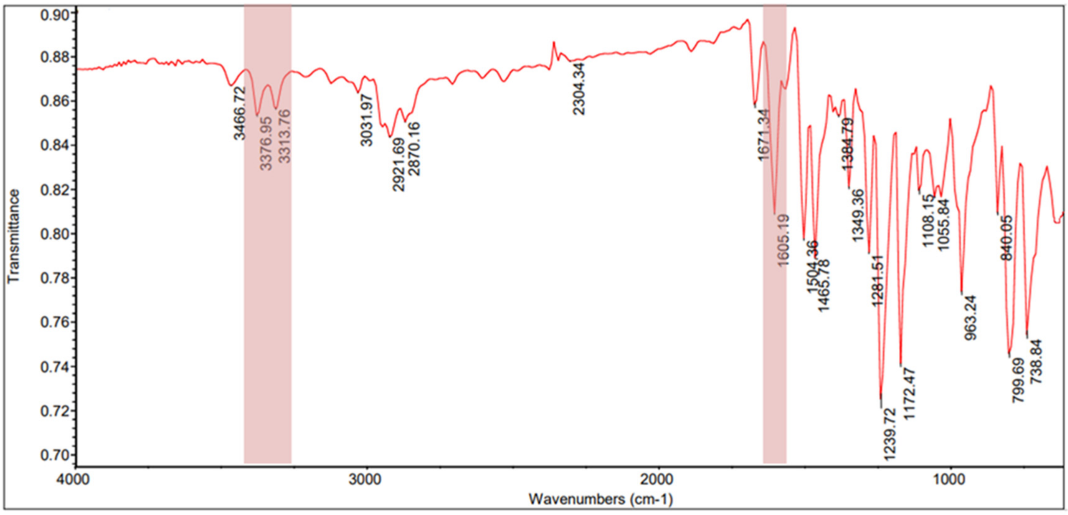

Figure S8. FTIR spectrum of compound **3**.

5-(4-Azidophenyl)-10,15,20-tris(4-(4-bromo-n-butoxy)phenyl)porphyrin **4**

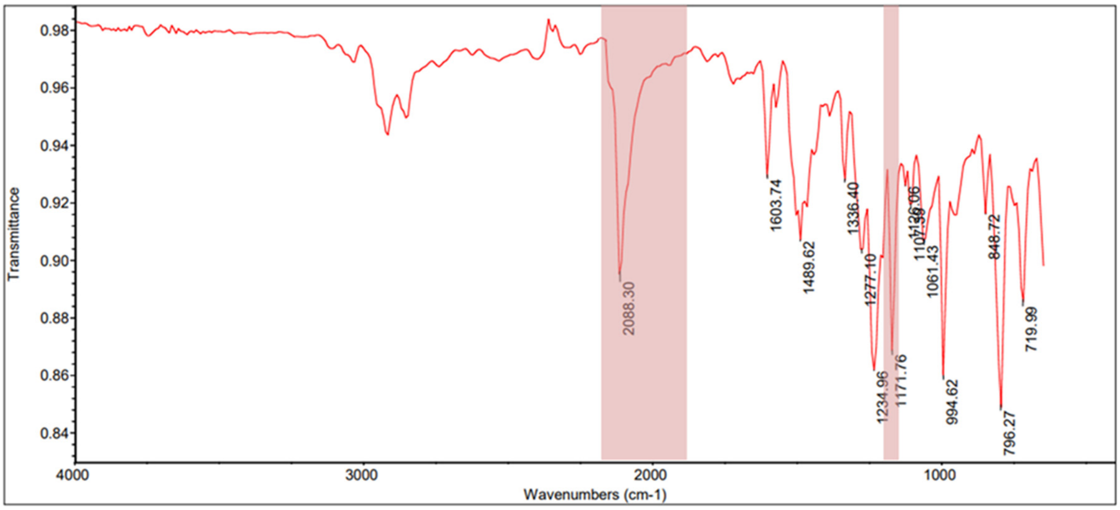

Figure S9. FTIR spectrum of compound **4.**

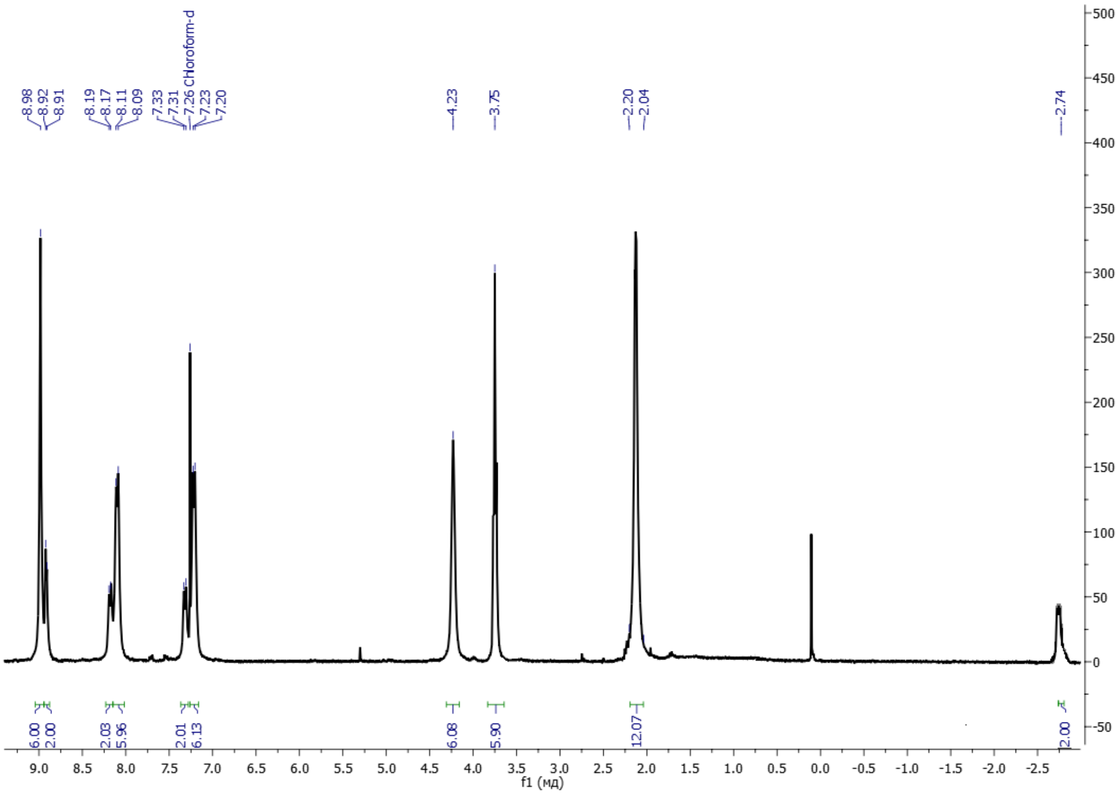

Figure S10. ^{1}H NMR of compound **4** ($CDCl_3$).

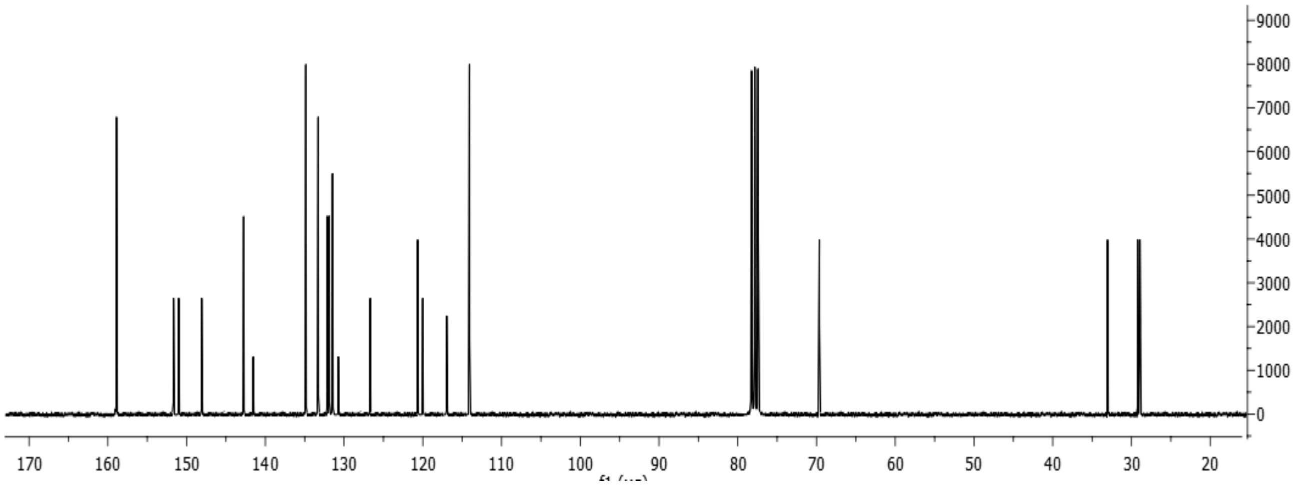

Figure S11. ^{13}C NMR of compound **4** ($CDCl_3$).

Zn (II) complex of 5-(4-azidophenyl)-10,15,20-tris(4-(4-bromo-*n*-butoxy)phenyl)porphyrin **5**.

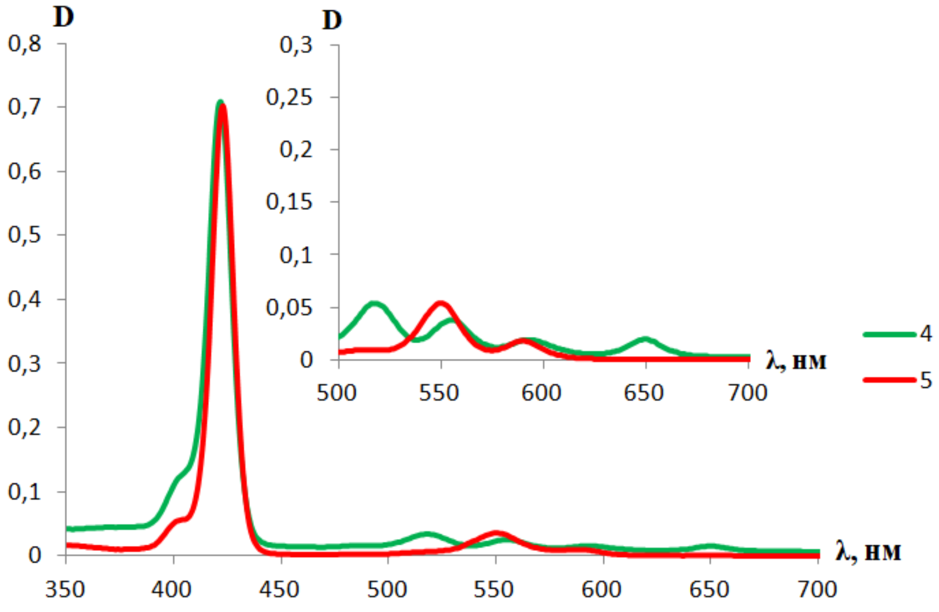

Figure S12. Electronic spectrum of compound **4** and **5** in CH_2Cl_2.

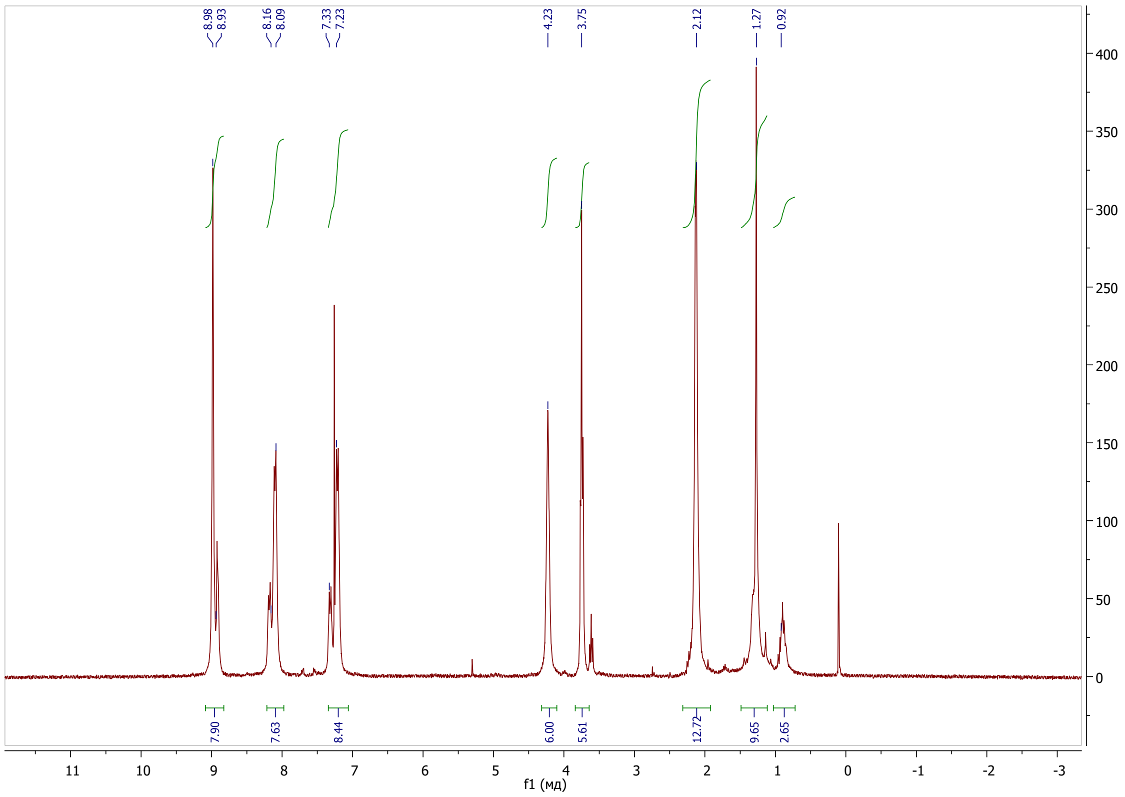

Figure S13. ^{1}H NMR of compound **5** ($CDCl_3$).

Conjugate **6.**

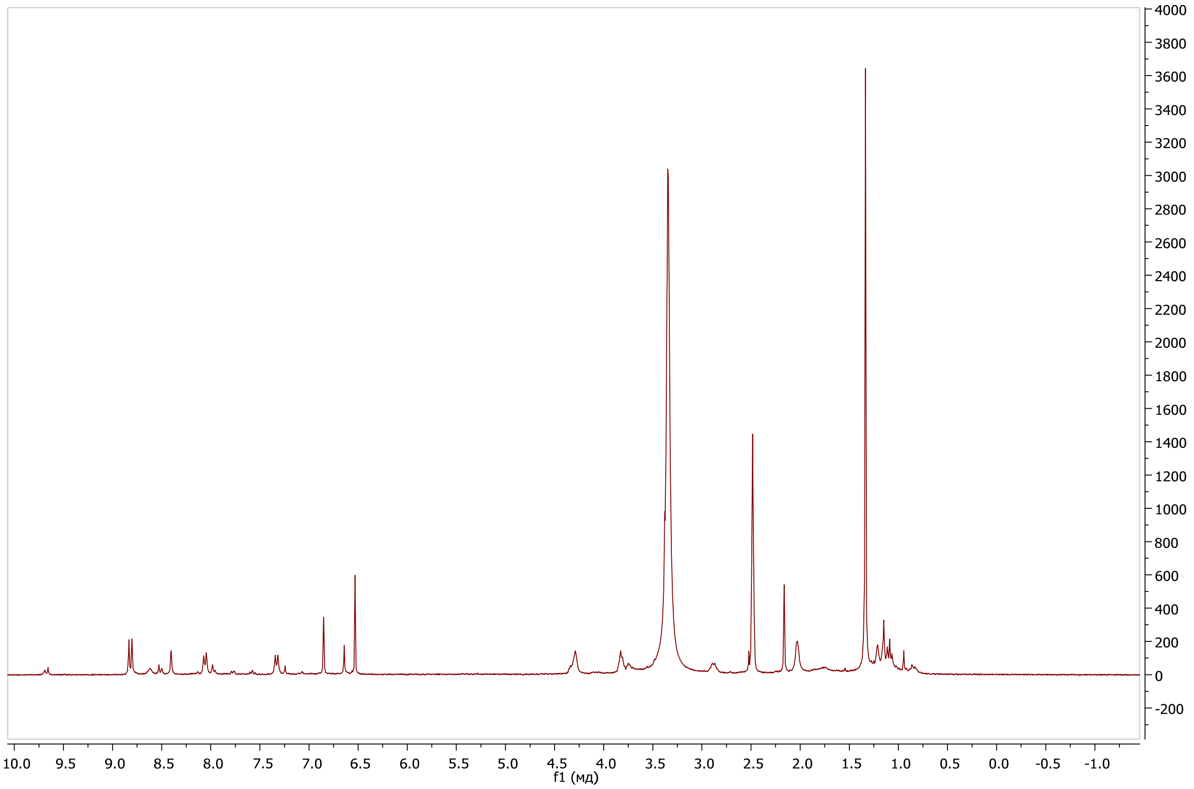

Figure S14. ^{1}H NMR of compound **6** ($CDCl_3$).

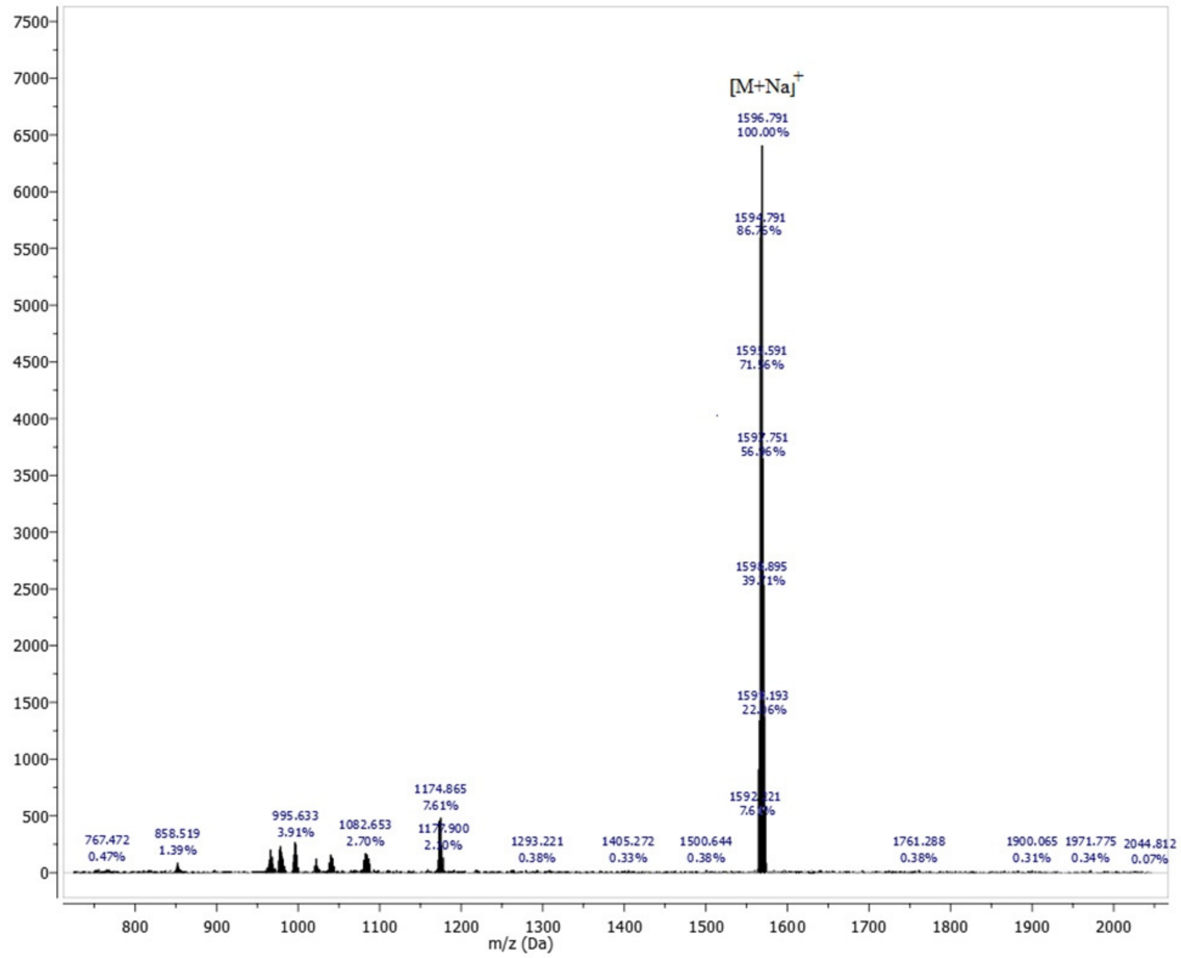

Figure S15. MALDI-TOF mass-spectrum of compound **6**.

Conjugate **7.**

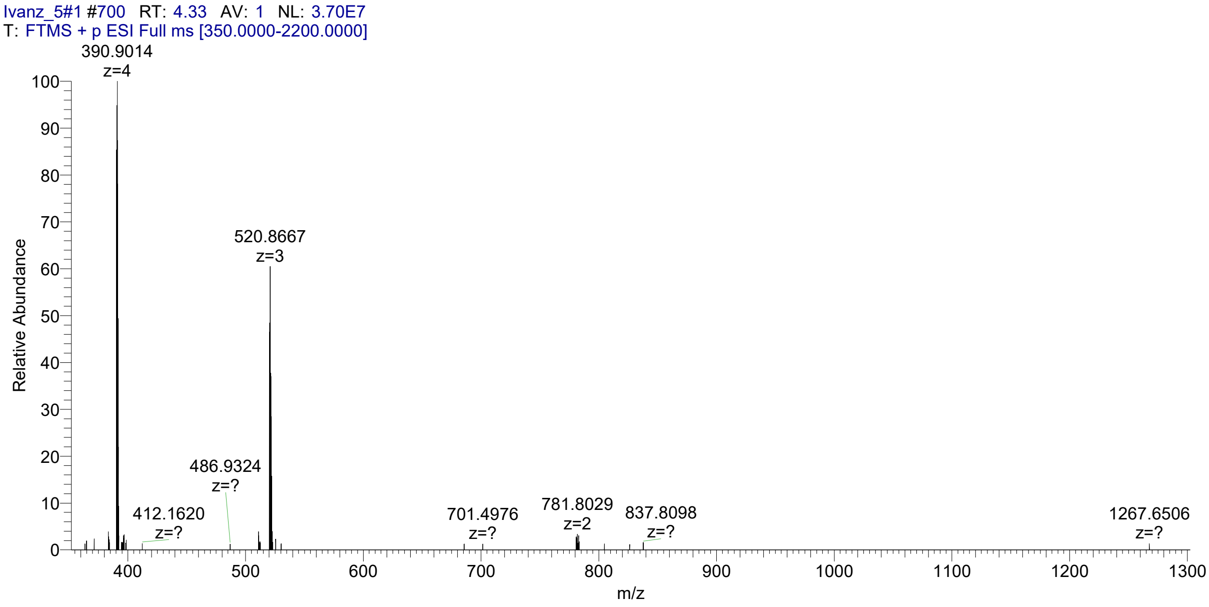

Figure S16. ESI- HRMS mass spectrum of **7** m/z $[M+H]^{3+}$ = 520.8666.

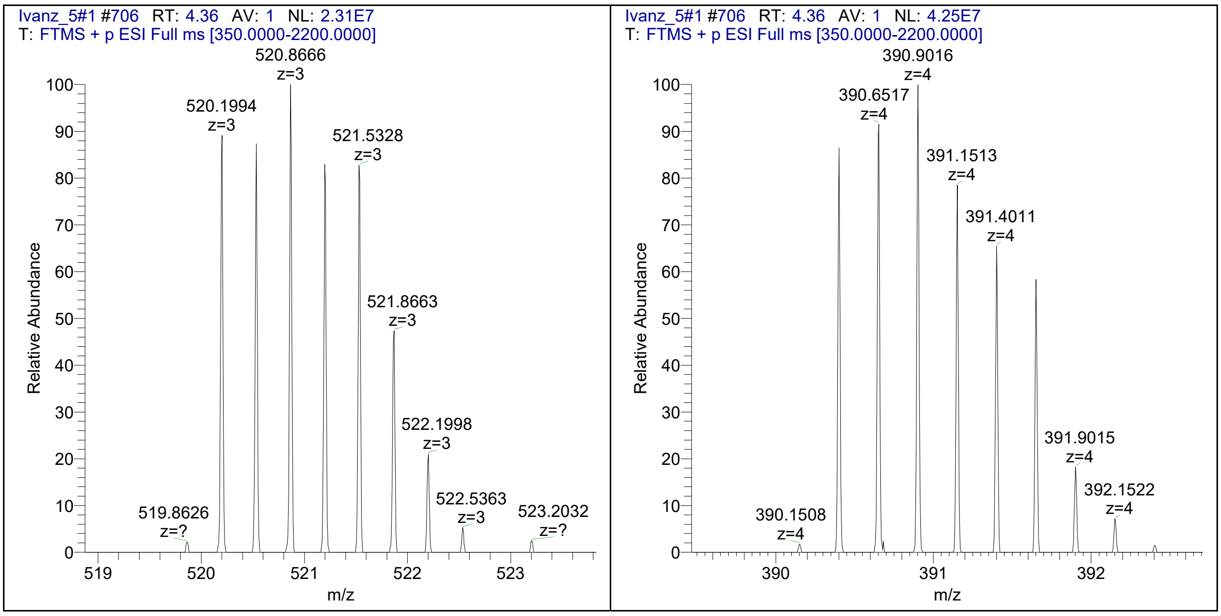

Figure S17. ESI- HRMS mass spectrum of **7** m/z $[M+H]^{3+}$ = 520.8666.

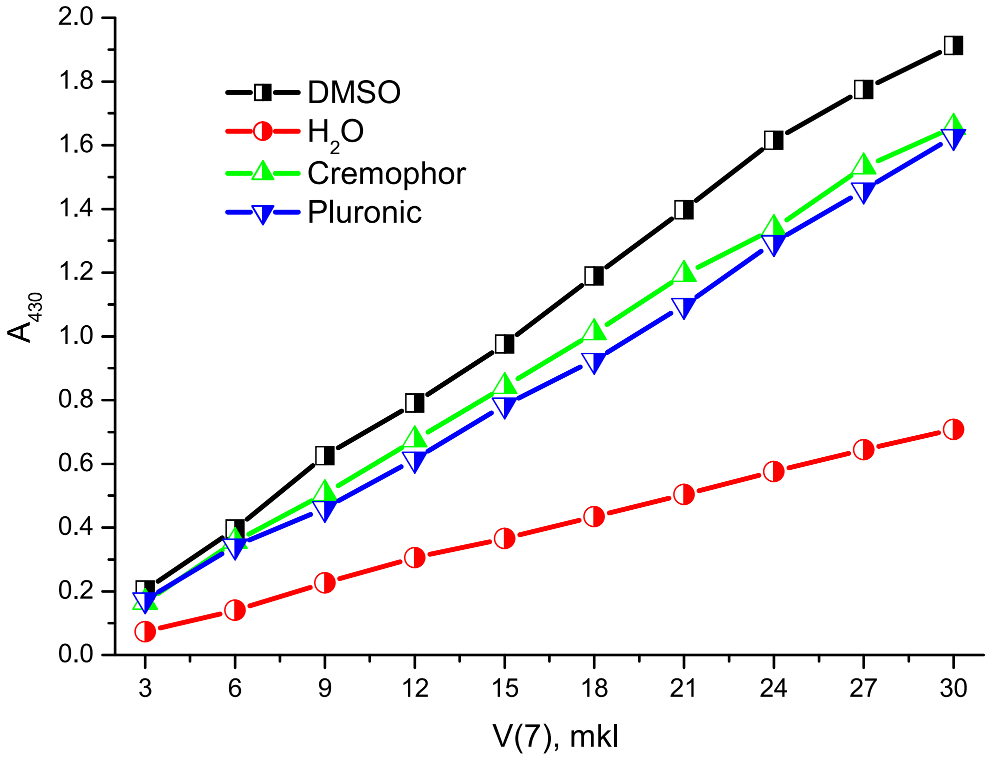

Figure S18. Concentration dependence of absorbance for 7 in different media.

Table S1. Particle size distribution by intensity for **7** in different media.

	Diameter, nm (Std. Dev.)			
	H_2O	DMEM	PBS	Pluronic
**Peak 1**	-	-	-	26 (2)
**Peak 2**	198 (45)	-	379 (69)	-
**Peak 3**	713 (141)	671 (167)	-	512 (118)

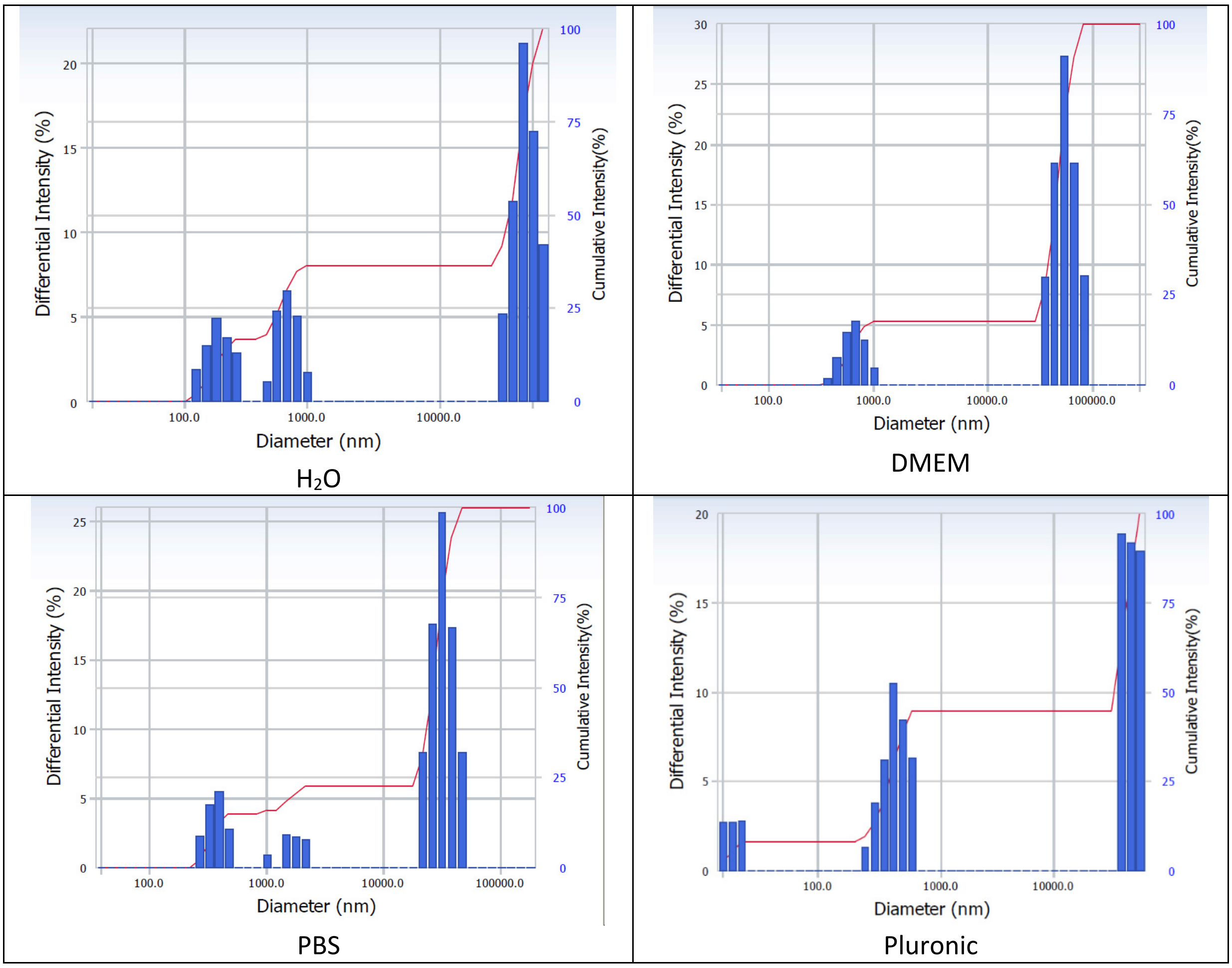

Figure S19. Particle size distribution by intensity for compound **7** in different media.
